# Design, spectral, molecular modeling, antimitotic, analytical and mechanism studies of phenyl isothiocyanate Girard's T derived metal complexes

**DOI:** 10.1186/s13065-023-01033-x

**Published:** 2023-11-12

**Authors:** Magda A. Akl, Nora A. El Mahdy, Zizi Elbadrawy, Abdelrahman S. El-Zeny, Mohsen M. Mostafa

**Affiliations:** Chemistry Department, Faculty of Science, Mansoura, University, Mansoura, 35516 Egypt

**Keywords:** Cu(II), Co(II), Ni(II), Girard-T, DFT calculations, Molecular docking, Cytotoxic activities, Flotation

## Abstract

The ligand N-{[(phenyl amino) thioxomethyl] hydrazino carbonyl methyl} trimethyl ammonium chloride (PTHAC) was prepared by the refluxing phenyl isothiocyanate and Girard-T (trimethyl ammonium-acethydrazide) in a molar ratio (1:1). The metal complexes derived from NiCl_2_.6H_2_O, CuCl_2_.2H_2_O and Co(CH_3_COO)_2_.6H_2_O were synthesized and purified. The PTHAC and its Cu(II), Co(II), and Ni(II) metal complexes(**1–3**) were characterized using a variety of various instrumental performances such as elemental analysis, magnetic moment, spectra (IR, UV–Vis, ^1^H NMR, mass) and thermal analysis (TGA and DTG).The results of element analysis, magnetic moment, spectra (IR, UV–Vis, ^1^H NMR, mass), and thermal (TGA and DTA) analyses provide the structures of the produced ligand and its (**1–3**) complexes. According to the spectroscopic results, PTHAC acts as an O, N and S tridentate donor, creating a mononuclear complex with copper(II), cobalt(II), and nickel(II) ions with an octahedral geometry. All of the atomic properties, including bond lengths, bond angles, HOMO, LUMO, dipole moments, and charges, have been determined. The cytotoxic activities of the PTHAC and the produced (**1–3**) complexes against breast carcinoma cells have been studied and correlated to the molecular modeling. When compared to the free ligand, CoII-L, and NiII-L, the CuII-L complex inhibits breast cancer cell growth more effectively. Furthermore, the PTHAC ligand was successfully applied for separation via flotation and spectrophotometric determination of Co(II) in several natural water, certified ore and pharmaceutical samples using oleic acid surfactant (HOL). At pH 6.5, PTHAC reacted with Co(II) to create a dark green (1:1) Co(II):PTHAC complex that was floated significantly using oleic acid (HOL) surfactant. The different experimental variable affecting the separation procedure e.g. pH, concentration of Co(II), HOL, PTHAC, temperature etc.…, were investigated. Co(II) had a linear range of (0.1–7.0) mgL^−1^. In the aqueous and scum layers, the molar absorptivities for the coloured complex are 0.14 × 10^4^ and 0.16 × 10^5^Lmol^−1^ cm^−1^, respectively. The LOD was 0.04 mgL^−1^, which is related to Sandell sensitivity of 3.7 × 10^−3^ µg cm^−2^ with a preconcentration factor of 200 and a RSD, % (n = 5) less than 4.2%. In addition, the mechanisms involved in the process of coordination of PTHAC with Cu(II), Co(II) and Ni(II) and the mechanism involved in the process of flotation of the PTHAC-Co(II) complex using HOL surfactant were elucidated.

**Supplementary Information:**

The online version contains supplementary material available at 10.1186/s13065-023-01033-x.

## Introduction

Many different organic compounds act as reagents for the determination of the functional groups of other molecules [1, 2]. One significant class of these reagents is the Girard reagents which create water-soluble hydrazones and are used to isolate carbonyl compounds from complex compounds. In 1936, the Girard-T and Girard-P reagents were generated [3]. These two reagents are water-soluble, and research indicates that they block the activity of specific enzymes (histidine decarboxylase [4], acetylcholine esterase[5], and aryl sulfatase [6, 7]. Both Girard-T and Girard-P reagents have been used to facilitate several separation techniques [1, 2, 8–10], and this is how Lehn et al.[11] employed them as platforms for the gradual release of fragrant aldehydes. More and more studies have been carried out in recent years to determine whether or not the anti-tumor properties of inorganic, and especially metal complexes, may be used to treat cancer disorders. Researchers have developed novel platinum complexes and complexes including metals such as gold, copper, iron, vanadium, cobalt, ruthenium and manganese in response to the success of cisplatin in clinical application for cancers [12]. Copper and gold complexes exhibit great action against many tumor systems, putting them the organometallic compounds with the greatest promising [13].

Complexes of copper(II) and copper(I) and Girard-T reagent of the formulas [Cu(HGT)Cl_2_(H_2_O)_2_]Cl⋅H_2_O⋅EtOH, [Cu(GT)(EtOH)_3/2_]Br_2_, [Cu(HGT)I_2_]⋅H_2_O and [Cu(HGT)I]I were reported by Mostafa and AbdelRhman [14]. The first was prepared by the reaction of ethanolic solutions of CuCl_2_⋅2H_2_O and the Girard-T reagent in the molar ratio of 1:2; and the second one by the reaction of the first complex with KBr. It is interesting to note that this tribochemical reaction of preparation of the bromide salt is accompanied by the deprotonation of the organic ligand. For both complexes, a bidentate NO coordination of the Girard-T reagent was proposed. The iodido complexes were also prepared by the tribochemical reaction of the mentioned chlorido complex and CaI_2_ or KI, and, due to the reducing properties of iodide, the product was a diamagnetic copper(I) complex. It is important to note that the newest evaluation of the biological activity of the above-mentioned chlorido complex showed that it is a promising antitumor agent [15].

The sulfonamide-derived metal complexes are substantial in line with their photochemical, photo-physical, biological and catalytic properties. The literature survey reveals that benzene sulfonamide established Schiff bases and metal complexes are imperative due to their biotic activities. As pharmacologically important possibilities, sulfonamide units are frequently used as antimicrobial, antioxidant and anticancer therapeutics as well as insulin mimics and enzyme inhibitors [16]. Many bioactive compounds have been developed as a result of their unique characteristics. Bearing in mind the above evidence, Hassan and Sumrra reported the synthesis, characterization, medicinal and molecular modeling of newly synthesized bidentate sulfonamide metal (VO^2+^, Co^2+^, Ni^2+^,Cu^2+^ and Zn.^2+^) chelates [17].

Aromatic rings form energetically favorable interactions with many polar groups in chemical and biological systems. Recent molecular studies have shown that sulfonamides can chelate metal ions and form hydrogen bonds. However, it is presently not established whether the polar sulfonamide functionality also interacts with aromatic rings. Hammink et al. reported synthetic, spectroscopic, structural and quantum chemical analyses on 2,6-diaryl benzene sulfonamides in which two flanking aromatic rings are positioned close to the central sulfonamide moiety. Fine tuning the aromatic character by substituents on the flanking rings leads to linear trends in acidity and proton affinity of sulfonamides. This physical-organic chemistry study demonstrated that aromatic rings have a capacity to stabilize sulfonamides via through-space NH-π interactions. These results have implications in rational drug design targeting electron-rich aromatic rings in proteins [18].

Determination of low-level analytes in a sample matrix when their concentrations are near the detection limit of the analytical equipment and where background interferences diminish the analytical signals is a major concern in environmental analysis. Several sample preparation and preconcentration methods have been developed to resolve this problem. The preconcentration methods are based on physical, physicochemical, and chemical principles [19–21]. Flotation techniques have many advantages over other concentration methods such as greater enrichment factors, the lack of emulsions, safety when dealing with potentially hazardous samples, low costs due to low reagent consumption, inexpensive instruments, flexibility and easier incorporation into automated analytical methods [22–26]. Ion flotation is one kind of flotation, while precipitate flotation is another (inorganic and organic).

In the past two decades, enzyme modeling and diagnostic devices have stimulated an increased interest in the production of metal complexes of polydentate N and S ligands [27, 28]. Because of their effective complexing characteristics towards heavy metals, the usage of these ligands was extended to environmental concerns.

To the best of our knowledge**,** no information was reported in the literature concerning phenyl isothiocyanate Girard-T metal complexes with Cu(II), Co(II), or Ni(II) in terms of characterization, spectroscopic, thermogravimetric, molecular modeling or cytotoxic activities [29].

The present study was carried out with the following objectives:

i. Synthesis and characterization of phenyl isothiocyanate Girard-T(PTHAC) metal complexes with Cu(II), Co(II) and Ni(II) using various instrumental performances as elemental analyses, magnetic moment, spectra (IR, UV–Vis, ^1^HNMR, mass), and Thermal analysis (TGA and DTG).
ii. Studying the molecular modeling of PTHAC derived- metal complexes.
iii. Studying the cytotoxic activities of the PTHAC and its metal complexes with Cu(II), Co(II) and Ni(II).
iv. Studying the optimum parameters of the flotation and FAAS determination of Co(II) using PTHAC ligand like pH, the concentration of metal and ligand,, temperature, flotation time, etc.
v. Elucidation of the mechanism of coordination of PTHAC with Cu(II), Co(II) and Ni(II).
vi. Elucidation of the mechanism of flotation of the PTHAC-Co(II) complex using HOL surfactant.

## Experimental

### Materials and instrumentation

All the chemicals were brought from Aldrich and Fluka and were used without additional purification. A series of standard solution were prepared by appropriate dilution of stock solution. An oleic acid (HOL) stock solution (6.36 × 10^−2^ moll^−1^) was prepared by dispersing 20 mL of HOL food grade (d 0.895), in 1 L kerosene. All other reagents used were of analytical reagent grade.

Carbon and hydrogen contents were determined using Perkin-Elmer 2408 CHN analyzer. The elemental analysis and some physical data of the free ligand and its metal complexes are reported in Table 1.The infrared spectra of the ligand and the isolated solid complexes were recorded as KBr discs on Mattson 5000 FTIR Spectrophotometer. The obtained spectral data are presented in Table 2. The mass spectra of the ligand and complex were recorded using Direct Inlet unit (DI-50) of Mass Spectrometer model SHIMADZU GC/MS-QP5050A. ^1^H-NMR spectra in d_6_-DMSO were used to record on a Varian-Hg VX-300 NMR spectrometer. The magnetic moment values were evaluated at room temperature using a Sherwood scientific magnetic susceptibility balance at 298 K. Molar conductance of the ligand and the metal complex (10^−3^ M) was applied in DMSO. The conductance values were recorded using Model 4510 Conductivity Meter RS 232. Thermal measurements (TGA, DTA) were recorded on a DTG-50 Shimadzu thermo-gravimetric analyzer at a heating rate of 10 °C/min and nitrogen flow rate of 20 ml/min. The chloride anion was determined gravimetrically as AgCl. The concentration of the separated Co(II) was determined using a Griffin Model 40 colorimeter and was confirmed by FAAS measurements at 283.3 nm with Perkin-Elmer 2380 atomic absorption spectrometer. The flotation cell was the same as previously specified [23]. It was a cylinder with an inner diameter of 15 mm and a length of 290 mm with a stopcock at the bottom and a stopper at the top. An electronic pH meter (Hanna Instruments model 8519) was used to measure the pH of sample solutions.

**Table 1:** Analytical data and some physical properties for ligand (PTHAC) and its metal complexes(1–3)

Cmpd.	Compound (Empirical formula)	M. wt	Colour	M. p (°C)	Analytical Found, Calculated%				μeff., B.M	A_m_^a^ in DMSO
No.					C	H	M	Cl		
**1**	[Cu(LH)Cl(EtOH)(H_2_O)]^1^/_2_EtOH^1^/_2_H_2_O [CuC_12_H_18_N_4_Cl_2_OS.1^1^/_2_(EtOH.H_2_O)]	497	Green	320	36.0 36.3	5.8 6.0	13.6 13	14.5 14.3	2.0	25
**2**	[Co(LH)AC(EtOH)(H_2_O)]EtOH [CoC_12_H_18_N_4_ClOS.AC.2EtOH.H_2_O]	530	Grey green	320	40.2 40.8	5.5 6.6	11.8 11.3	6.7 6.6	6.2	24.1
**3**	[Ni(L–H)EtOH)]Cl [NiC_12_H_18_N_4_Cl_2_OS.EtOH]	441.9	Orange	320	38.2 38.0	5.9 5.7	13.5 13.3	16.2 16.0	3.9	25.6

**Table 2:** Significant (IR) spectral bands (cm^−1^) of the ligand (PTHAC, L) and its metal complexes(1–3)

Compd	*V* (H_2_O)	*V(*NH^1^*)*	*V*(NH^2^)	*V*(NH^4^)	*V*(C = O)	*V*(C = N)	*V*(N–N)	*V*(C = S)	*V*(C-S)	*V*(M–O)	*V*(M–N)	*V*(M-S)
No.												
**L**	3422	3239	3191	4141	1704	-	920	718	–	–	–	–
**(1)**	3418	3062	–	3021	1736	1623	953	–	687	566	380	446
**(2)**	3407	3213	–	3054	1684	1670	923	–	676	524	369	439
**(3)**	3407	3200	–	3128	1667	1620	971	–	679	529	372	436

### Preparations

#### Preparation of ligand

**Scheme 1:**
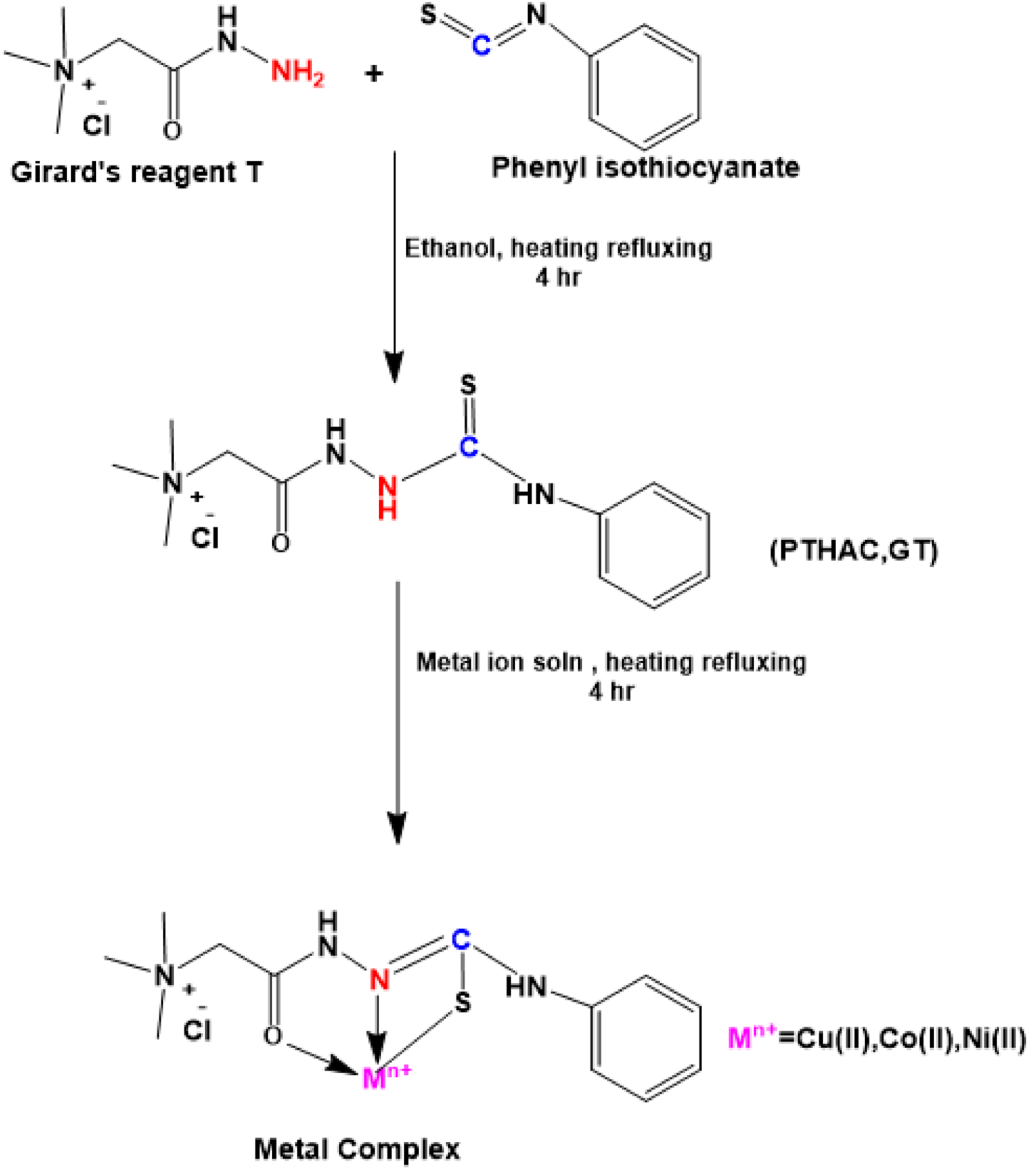
Preparation of the ligand (PTHAC) and its metal complexes

The ligand N-[(phenylamino) thioxomethyl] hydrazino carbonyl methyltrimethyl ammonium chloride (PTHAC) [19], Scheme 1, was prepared by mixing GT (16.8 g, 0.1 M) and phenyl isothiocyanate (12 ml, 0.1 M) in 100% ethanol (100 ml) and heating the mixture under reflux for 4 h. Hot absolute ethanol solution and dry diethyl ether were used to wash and filter the white precipitate. The isolated product was dried in an oven at 90 °C, crystallized from absolute ethanol, and then dried in a vacuum desiccator over silica gel. The purity of the isolated compound was investigated with m.p., TLC, and molecular weight measurements.

#### Preparation of the solid complexes

##### In aqueous ethanol solution

In this investigation, the complexes of CuCl_2_.2H_2_O, CoCl_2_.2H_2_O, Co(CH_3_COO)_2_.6H_2_O and NiCl_2_.6H_2_O were prepared by mixing their solutions in absolute ethanol with the calculated amounts of the investigated ligands in 1:1, 1:2 molar ratio. These mixtures were heated under reflux for about 2 h. The pH of these mixtures was found to be 5.5. The complexes thus formed was filtered off, recrystallized from ethanol and finally dried in an oven at about 120 ºC and the purity was checked by TLC.

The precipitates were filtered off and dried in vacuum desiccators over anhydrous phosphorous pentaoxide (P_4_O_10_).

##### In the scum layer

Isolated Co(II) complex from the scum was formed by combining ligand and Co(II) ions at a concentration of (1 × 10^−2^moll^−1^, each) in the presence of (6.36 × 10^–2^ moll^−1^) oleic acid in a volume of 3 ml. The float (solid complex) was collected by filtration and rinsed several times with ethanol and diethylether. The precipitate was dried in an oven at 80 °C and kept in a desiccator.

### Molecular modeling

The structures of the PTHAC ligand and its Co^2+^, Ni^2+^ and Cu^2+^complexes were obtained using generalized gradient approximation (GGA) with DFT calculations using the DMOL^3^ tool in the Material Studio (MS) package, with optimized BLYP correlation function method and Double Numerical polarization basis-set (DNP). Vibrational frequency calculations were performed to ensure that each structure had the lowest potential energy surface. The highest occupied molecular orbital (HOMO) and lowest unoccupied molecular orbital (LUMO) are very important factors in theoretical molecular design [30]. HOMO is the electron donor and the LUMO is the electron acceptor site. The molecular hardness and softness of the compound can be predicted from its HOMO–LUMO gap. HOMO/LUMO energies were estimated at the DFT/BLYP level and optimized geometries were obtained.

The electronic properties and reactivity definers such as ionization potential (IP), electron affinity (EA), hardness (η), softness (σ), electronegativity (χ), electrophilicity index (ω), and chemical potentials (µ)] can be determined from the HOMO and LUMO orbital energies through Koopman’s theorem. The energy gap (ΔE), electronegativity ($\chi$), hardness ($\eta$), chemical potentials (µ), softness ($\sigma$), and electrophilicity index (ω) were calculated using subsequent equations, Table 3 [31, 32]

**Table 3:** Global Chemical Reactivity Descriptors(**G**C**R**D)

GCRD	Equation
The energy gap (ΔE_gap_)	ΔE_gap_ = (E_LUMO_ – E_HOMO_)
Ionization potential (I_P_)	I_P_ =−E_HOMO_
Electron affinity (E_A_)	E_A_ =−E_LUMO_
Hardness (η)	$\eta =\frac{{I}_{P}- {E}_{A}}{2}$
Softness (${\varvec{\sigma}}$)	$\sigma =\frac{1}{\eta }$
Electronegativity (χ)	$\chi =\frac{{I}_{P }+{ E}_{A}}{2}$
Chemical potentials (µ)	µ = −$\chi$
The fraction of electron transferred (ΔN)	(ΔN)$=\frac{{\upchi }_{\mathrm{Ce }}-{\upchi }_{\mathrm{Ligand}}}{{2(\upeta }_{\mathrm{Ce }}+{\upeta }_{\mathrm{Ligand}})}$
Electrophilicity index (ω)	(ω) = $\frac{{\upmu }^{2}}{2\eta }$
The back donation	(ΔE_back-donation_)$=-\frac{\upeta }{4}$

### Estimation of the antitumor activity

The MFC-7 cell lines were obtained from the American type Culture Collection (ATCC, Rockville, MD). The cells were grown on RPMI-1640 medium supplemented with 10% inactivated fetal calf serum and 50 µg/ml gentamycin. The cells were maintained at 37 °C in a humidified atmosphere with 5% CO_2_ and were subcultured two to three times.

MCF-7 cancer cell lines were applied to evaluate the antitumor activity et al.-Azhar University's Regional Institute for Mycology & Biotechnology in Cairo, Egypt. The cell lines were cultured as monolayers in growth media containing 10% acellular foetal calf serum and 50 µg/ml gentamycin. Ten thousand cells were seeded into each well of a 96-well microtiter plate (Falcon, NJ, USA) and left to form monolayers at the bottom of the plates for 24 h at 37 °C in a humidified incubator with 5% CO_2_. After washing the monolayers with phosphorus saline (0.01 M, pH 7.2), the cells were treated with 100 µl from various dilutions of tested substance in new maintenance media and incubated at 37 °C. We also created a set of control cells that had not been treated with the tested compounds under investigation. Each concentration of test sample was applied to three separate wells. Every 24 h, samples were examined using an inverted microscope. Cells were stained with crystal violet, lysed with 33% glacial acetic acid, and their absorbance at 590 nm was examined using an ELISA reader to estimate how many cells had survived. The percentage of viability was determined by subtracting the mean optical density of wells treated with the tested compounds from that of wells left untreated; this was done by using the formula [1-(ODt/ODc)] × 100%, where ODt is the mean optical density of wells treated with the tested compounds and ODc is the mean optical density of wells left untreated [33–35].

### Molecular docking

Theoretical calculations like molecular docking can be used to aid scientists in drug design and discovery to propose the drug interaction model and give information about the behavior of new drugs toward the biological targets. The Protein Data Bank structure of breast cancer (PDB ID: 1jnx) was downloaded from (http://www.rcsb.org.pdb). Discovery studio was used to prepare the proteins for docking by removing small molecules and water molecules (> 5A radius) from the structure as well as the addition of hydrogen and disulphide bonds to PDB. The energy of the protein molecules and the coordination compounds were minimized using the energy minimization algorithm of Molecular Operating Environment (MOE2022 software). Then, the ligand L &its (Cu, Co, Ni) complexes ware prepared for docking. The energy of the ligand molecule and the coordination compounds were minimized using the energy minimization algorithm of Molecular Operating Environment (MOE2022 software). The binding of the ligand molecule with the protein molecule was analyzed using MOE docking program to find the correct conformation (with the rotation of bonds, structure of molecule is not rigid) [36, 37].

### Separation via flotation and spectrophotometric determination of Co(II)

Co(II) and the ligand (PTHAC) were mixed in a 3 ml of distilled water at the concentrations indicated for each trial. In order to get best results, the pH level was optimized. The solution was thereafter introduced into the flotation cell and brought to the required volume (20 ml) using bidistilled water. For a few seconds, the cell was vigorously shaken to achieve full complexation. Then, 3 ml of HOL (at a known concentration) were added to the mixture. A total of twenty manual inversions were performed on the flotation cell. After 5 min, to ensure full flotation, the metal ion concentration in the mother liquid or in the scum was measured spectrophotometrically or by FAAS.

The concentration of Co(II) in the mother liquor was used to determine the floatability (F%) of Co(II) using the relation:

$$\mathrm{F}=\frac{(\mathrm{Ci }-\mathrm{ Cf})}{\mathrm{Ci }}\times 100$$

where C_i_ and C_f_ represent the initial and final concentration of Co(II), respectively.

Alternatively, the scum layer was eluted using (3 ml M HCl + 1 ml M HNO_3_) and then the analyte concentration was assessed spectrophotometrically or by FAAS to determine the flotation efficiency with the formula:

$$\mathrm{F}=\frac{\mathrm{CS}}{\mathrm{Ci }}\times 100$$

where C_i_ and C_S_ represent the initial and final concentrations, respectively.

## Results and discussion

### Characterization

#### Elemental analysis

The ligand N-{[(phenyl amino) thioxomethyl] hydrazino carbonyl methyl} trimethyl ammonium chloride (PTHAC) was prepared by the condensation of phenyl isothiocyanate and Girard’s reagent T(trimethyl ammonium-acethydrazide) in a molar ratio (1:1). The metal complexes derived from NiCl_2_.6H_2_O, CuCl_2_.2H_2_O and (CH_3_COO)_2_Co.4H_2_O were synthesized and purified. The elemental analyses and some physical data of the free ligand and its metal complexes are reported in Table 1. Once cooled, both the ligand and the metal complexes are sparingly soluble in water and hardly soluble in ethanol, DMF, and DMSO. As it is completely soluble on hot DMSO which gives the opportunity to measure conductance and ^1^H-NMR but insoluble in other organic solvents Additional file 1: Table S1. The suggested structures of the isolated compounds are elucidated by elemental analyses, spectral (IR, ^1^HNMR, electronic and mass), conductance, and magnetic moment and TGA measurement.

#### ^1^HNMR

**Fig. 1:**
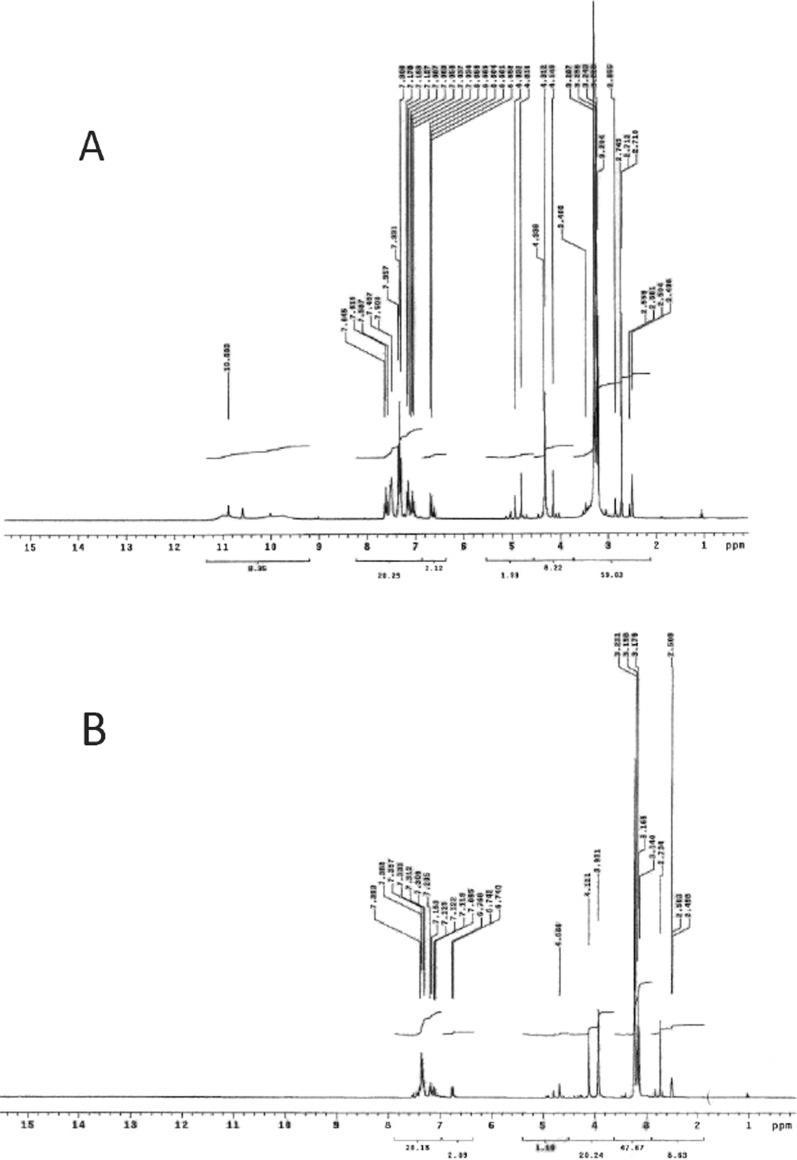
^1^HNMR spectrum of the ligand (PTHAC) in **A** d_6_-DMSO and **B** D_2_O

The ^1^HNMR spectra of the free ligand (PTHAC) have been recorded in d6-DMSO, Fig. 1. There are three signals in the ligand's ^1^HNMR spectra at δ 10.86, 10.58, and 10 ppm relative to (NH^1^), (NH^2^), and (NH^4^), respectively; they erase when D_2_O is added. These frequencies are correlated with protons and neutrons, respectively [38]. Multiple signals between δ 7.03 and 7.64 ppm are attributable to the proton in the phenyl ring [39]. At a frequency of δ 4.338 ppm, protons from CH_2_ are detected [40]. The (CH_3_)_3_ signals are located between δ 3.226 and 3.287 ppm [41]. In the ^1^HNMR spectrum of Cu(II) complex, new signals appear at δ 2.52, 3.37 and 12.22 ppm. These signals are attributed to CH_3_, CH_2_ and OH of the coordinated ethanol molecules.

#### FTIR spectra and mode of bonding

In order to ascertain the mode of bonding of the ligand, the IR spectrum of the free ligand is compared with the IR spectra of its metal complexes, Table 2**.**

**Fig. 2:**
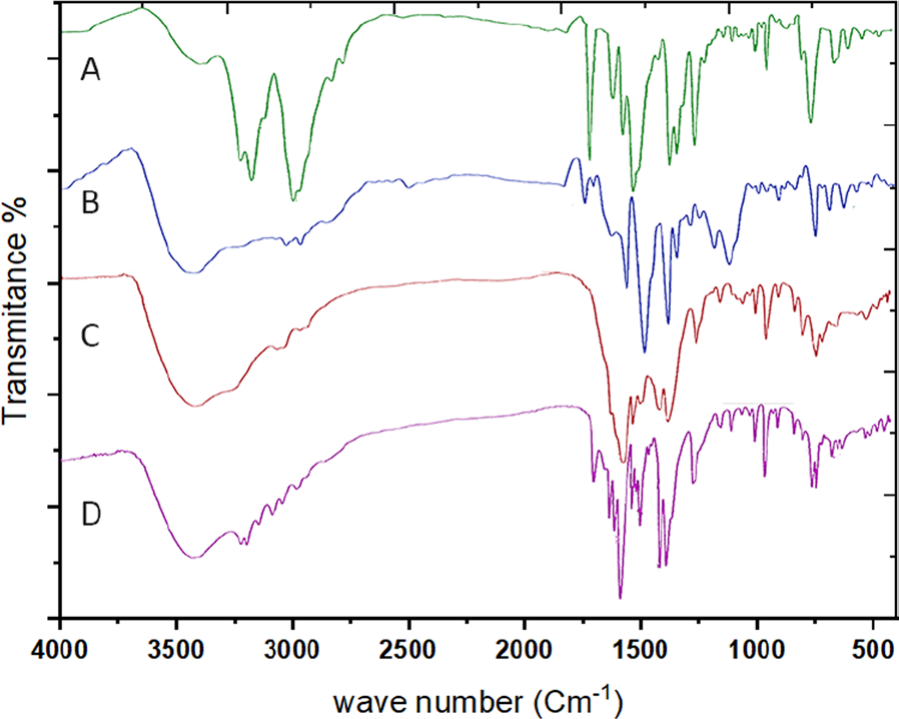
IR spectra of **A** ligand (PTHAC); **B** [Cu(L–H)Cl(EtOH)(H_2_O)].½EtOH.½ H_2_O; **C** [Co(L–H)Ac(EtOH)(H_2_O)].EtOH; **D** [Ni(L–H)EtOH]Cl

Figure 2A–D and Additional file 1: Figs. S1–S4 show the IR spectra of the free ligand and its metal complexes. Bands at 3239, 3191, and 3141 cm^−1^ in the IR spectrum of the ligand PTHAC, Fig. 2A, are attributed to the υ (NH)^1^[42], υ (NH)^2^[43], and υ (NH)^4^vibrations, respectively [44]. At 1704 cm^−1^, there is a strong band and a shoulder and this is assigned to (C = O) [40, 45]. The three strong bands at 1605, 1563 and 1513 cm^−1^ are assigned to the aromatic υ(C = C) vibrations [46]. The medium intensity bands at 1353, 1322 and 1299 cm^−1^ are attributed to υ(C-N). The strong band at 920 cm^−1^ is assigned to the υ(N–N) vibration [47, 48]. The medium intensity band at 718 cm^−1^ is assigned to υ(C = S).

The hydrated nature of the ligand is supported by the observation of an extensive band at 3422–3396 cm^−1^. This band is mainly owing to υ(OH)_w_ of water molecule and the inter-molecular hydrogen bond. The band υ(C = S) disappears due to formation of the (C-S) in the coordination and formation of (C = N).

The appearance of weak bands in the IR spectra of the complexes at (372–396), (523–580) and (435–442) cm^−1^ is due to υ(M–N) [36], υ(M–O) and ʋ(M-S) [49] vibrations, respectively; these bands are absent in the free ligand.

The observation of broad bands in the (3450–3407) cm.^−1^ regions in the spectra of complexes is attributed to the υ(OH), ρr(H_2_O) and ρw(H_2_O) vibrations for the coordinated water [50].

The IR spectrum of the copper complex (Fig. 2B) displays new bands at 1623, 687 cm^−1^, respectively. The IR spectrum of the cobalt complex (Fig. 2C) gives new bands at 1670, 676 cm^−1^. In the IR spectrum of Ni(II) complex (Fig. 2D) new bands appeared at 1620, 697 cm^−1^due to sharing in coordination and the band υ(NH)^2^ disappeared due to formation of band (C = N).

By comparing the infrared spectra of the free ligand (PTHAC) with those of its metal complexes, it can be noticed that the ligand coordinates to the metal ions without being deprotonated. The ligand acts as a neutral tridentate through (C = O), (C = N) and (C = S), structures **I-III**.
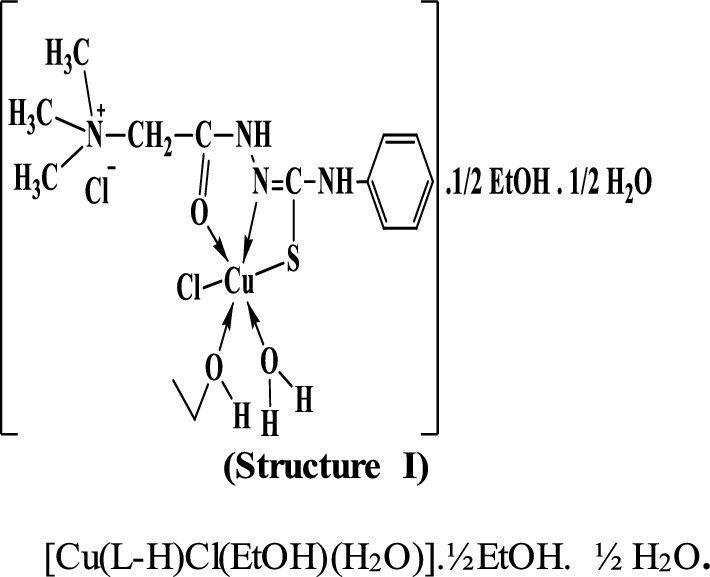



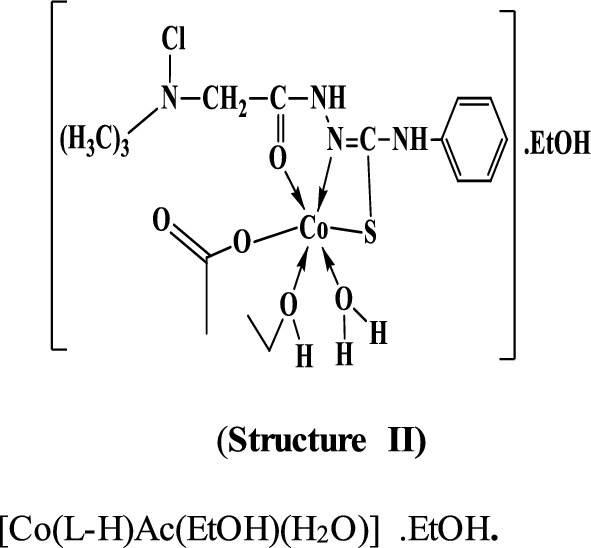



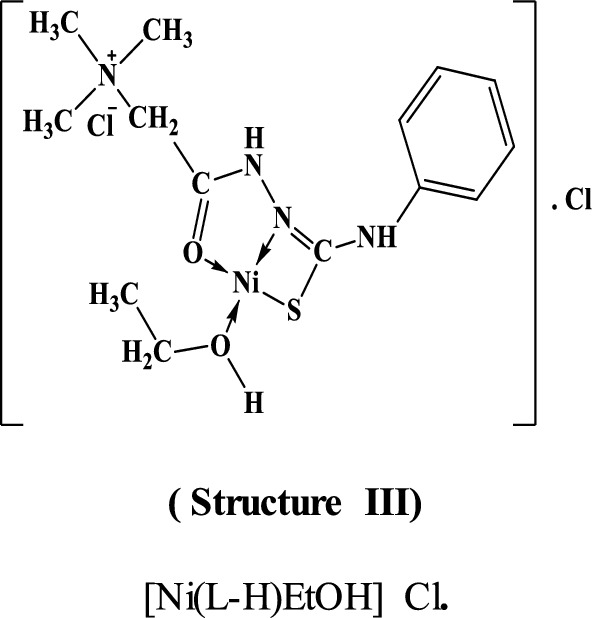


#### Mass Spectra

**Scheme 2:**
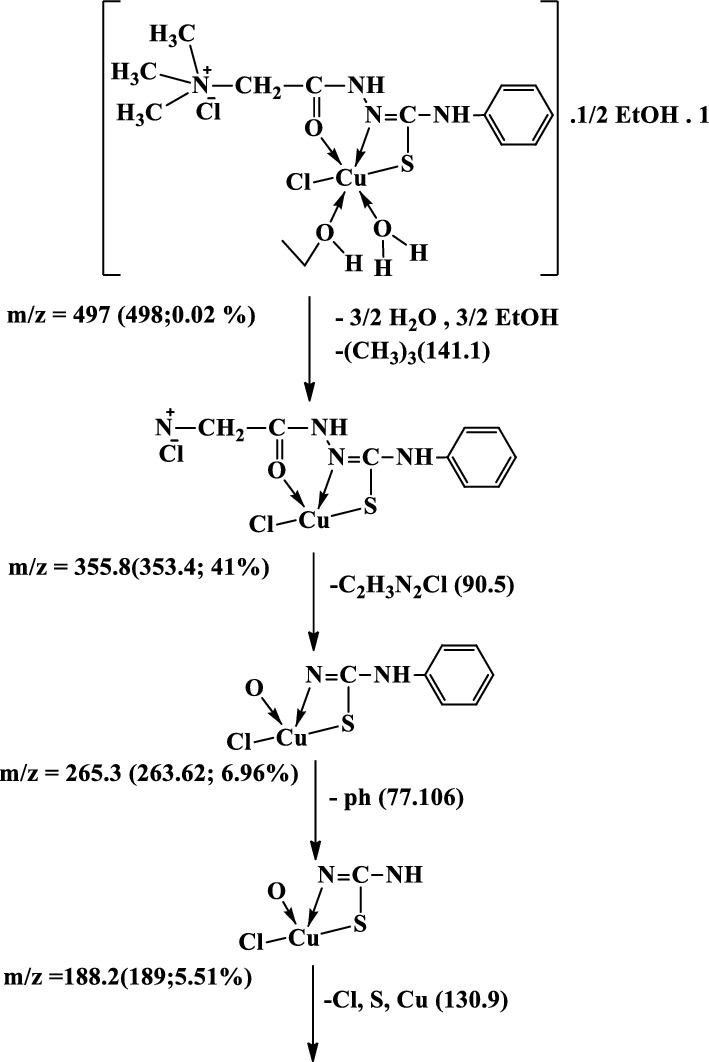
Mass fragmentation pattern of [Cu(L–H) Cl(EtOH)(H_2_O)].½EtOH.½H_2_O**(1)**

**Scheme 3:**
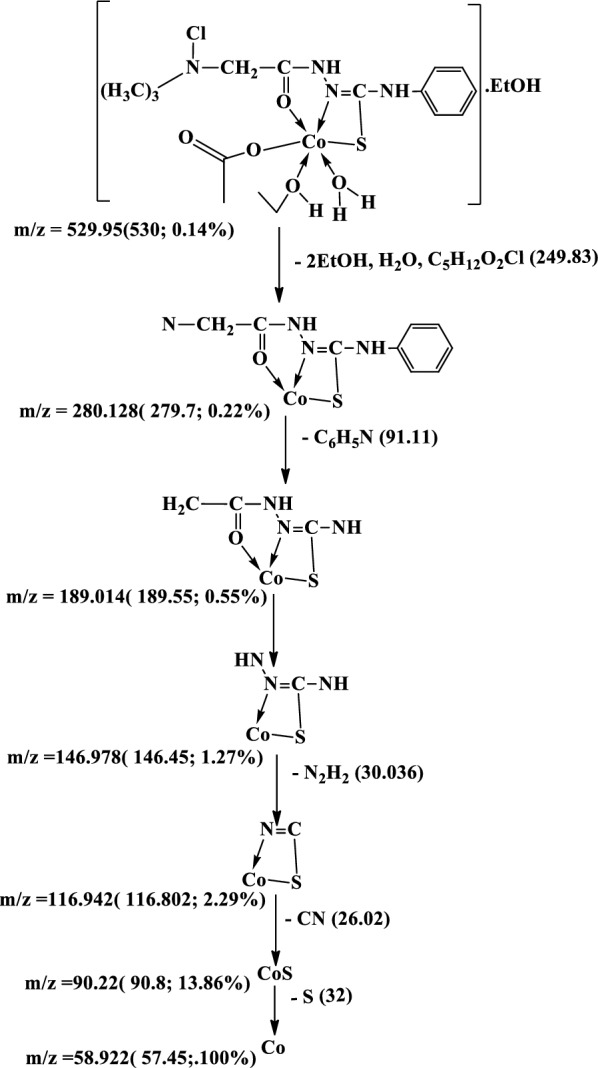
Mass fragmentation pattern of [Co(L–H)Ac(EtOH)(H_2_O)]. EtOH **(2)**

**Scheme 4:**
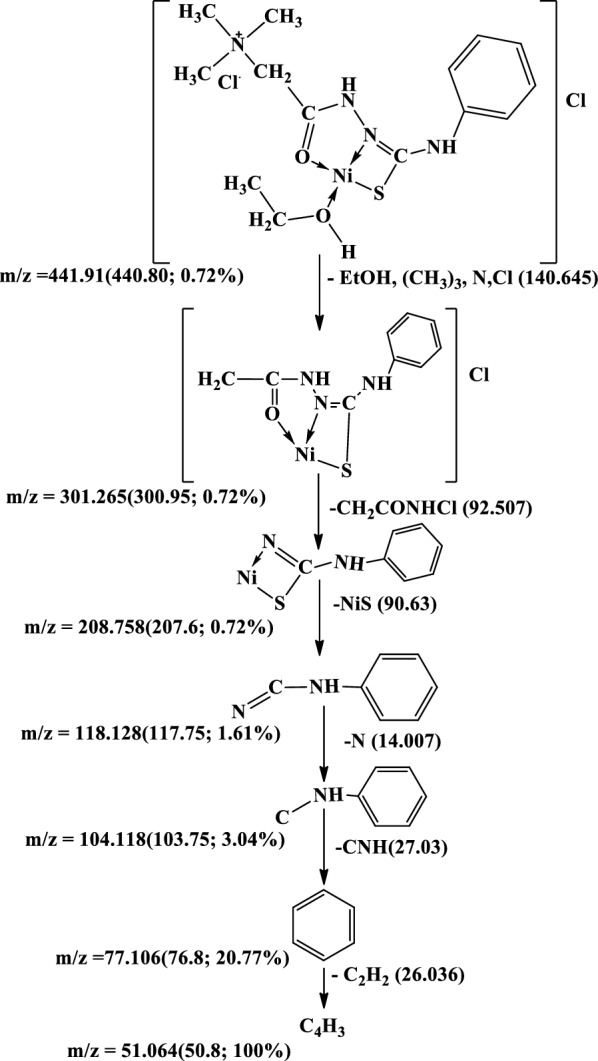
Mass fragmentation pattern of [Ni(L–H) EtOH] Cl **(3)**

The mass spectra of the Cu(II), Co(II) and Ni(II) complexes **(1–3)**, Additional file 1: Fig. S5a-c show the molecular ion peaks at m/z = 498, 530, 516 and 441 which agree with the molecular formula [CuC_12_H_18_N_4_Cl_2_OS.1½(EtOH.H_2_O)];497,

[Co C_12_H_18_N_4_ClOS.Ac.2EtOH.H_2_O]; 529.96, and [NiC_12_H_18_N_4_Cl_2_OS.EtOH]; 441.91, respectively. In addition, the mass spectra of the Cu(II), Co(II) and Ni(II) complexes show also peaks at 309, 314, 301 and 301 due to the fragmentation of the ligand (L), respectively. Schemes 2, 3, 4 show the proposed fragmentation patterns of the three complexes.

#### Molar conductivity measurements

The molar conductivity of the free ligand and its metal complexes, **(1–3)** was determined using 10^–3^ M solution of DMSO at room temperature (18 °C). The value of molar conductivity of the ligand (L) is 31.1 Ω^−1^cm^2^mol^−1^; this suggests the electrolytic nature and the existence of chloride ion inside or outside the coordination sphere [46].

The molar conductivity measurements of the metal complexes reveal the electrolytic nature of these complexes which is in agreement of the proposed structures, Table 4**.**

**Table 4:** Electronic spectral and magnetic moments of the ligand (L; PTHAC) and its metal complexes(**1–3**)

Compd.	μ_eff_ (BM)	State	Absorption bands (nm)		
No.			Π–π^*^	n–π^*^	d-d transitions
**L**	–	Nujol	240 nm, (41,667 cm^−1^) 272 nm, (36,765 cm^−1^) 292 nm, (34,246 cm^−1^) 341 nm, (29,326 cm^−1^)	–	–
**(1)**	2.0	Nujol	–	–	640 nm, (15,625 cm^−1^)
**(2)**	6.2	Nujol	304 nm,(32,895 cm^−1^) 338 nm,(29,586 cm^−1^)	412 nm,(24,272 cm^−1^)	614 nm,(16,287 cm^−1^) 676 nm,(14,793 cm^−1^)
**(3)**	3.9	Nujol	258 nm,(38,760 cm^−1^) 284 nm,(35,211 cm^−1^) 318 nm,(31,447 cm^−1^) 358 nm,(27,933 cm^−1^) 398 nm,(25,126 cm^−1^)	420 nm,(23,810 cm^−1^) 468 nm,(21,368 cm^−1^) 504 nm,(19,841 cm^−1^) 536 nm,(18,657 cm^−1^)	708 nm,(14,124 cm^−1^)

#### Electronic spectra and magnetic moment data

**Fig. 3:**
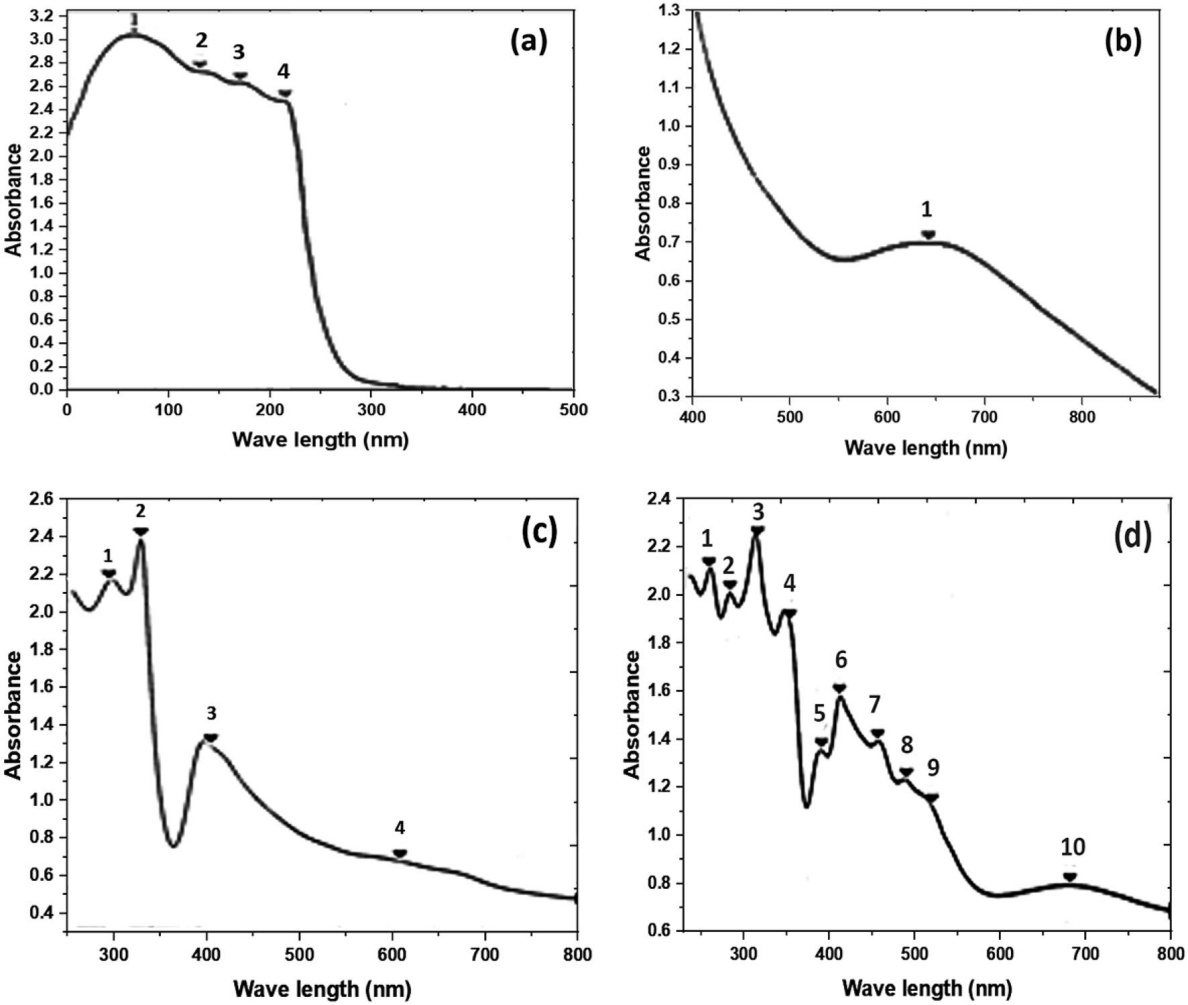
**a** Electronic spectrum of the ligand (L;PTHAC); **b** Electronic spectrum of [Cu(L–H)Cl(EtOH)(H_2_O)].½EtOH.½ H_2_O in Nujol **(1)**; **c** Electronic spectrum of [Co(L–H)Ac(EtOH)(H_2_O)]. EtOH in Nujol **(2)**; **d** Electronic spectrum of [Ni(L–H)Cl EtOH] in Nujol **(3)**

The electronic spectrum of the free ligand, Fig. 3a**,** shows four bands at 240, 272, 292 and 314 nm assigned to the π → π* transition of the phenyl ring, (C = N), (C = O) and (C = S) groups, respectively.

Figure 3b–d displays the electronic spectra of the metal complexes. Table 4 lists the value of the magnetic moments for the metal complexes.

Figure 3b shows the electronic spectrum of the Cu^2+^ complex, [Cu(L–H)Cl(EtOH)(H_2_O)].½EtOH.½H_2_O. The spectra of Nujol reveals a strong band at 15,625 cm^−1^ that corresponds to the ^2^T_2g_→^2^E_g_ transition in an octahedral geometry around the Cu^2+^ ion [51]. The magnetic moment value of [Cu(L–H)Cl(EtOH)(H_2_O)].½EtOH.½ H_2_O complex **(1)** is 2.0 BM.

The electronic spectrum of the [Co(L–H)Ac(EtOH)(H_2_O)].EtOH complex **(2)** in Nujol, Fig. 3c**,** exhibits two strong absorption bands at 16,287 and 14,793 cm^−1^ attributable to ^4^T_1g_^4^T_1g_ (P) and ^4^T_1g_→^4^A_2g_ transitions in a high-spin octahedral environment around the Co^2+^ ion [52]. The magnetic moment value (6.2 BM) confirms the presence of three unpaired electrons with no orbital contribution.

The electronic spectrum of the Ni^2+^ complex, [Ni(L–H)EtOH]Cl **(3),** in Nujol, Fig. 3d, shows two weak bands at 19,841 and 18,657 cm^−1^ which are attributed to spin-forbidden while the weak band at 14,124 cm^−1^ (708 nm) is assigned to ^3^T_1_→^3^T_1_ (P) in a tetrahedral geometry around the Ni(II) ion [53]. The value of magnetic moment (3.9 BM) is taken as an additional evidence of tetrahedral geometry around the Ni.^2+^ ion [54].

#### Thermogravimetric analysis

**Fig. 4:**
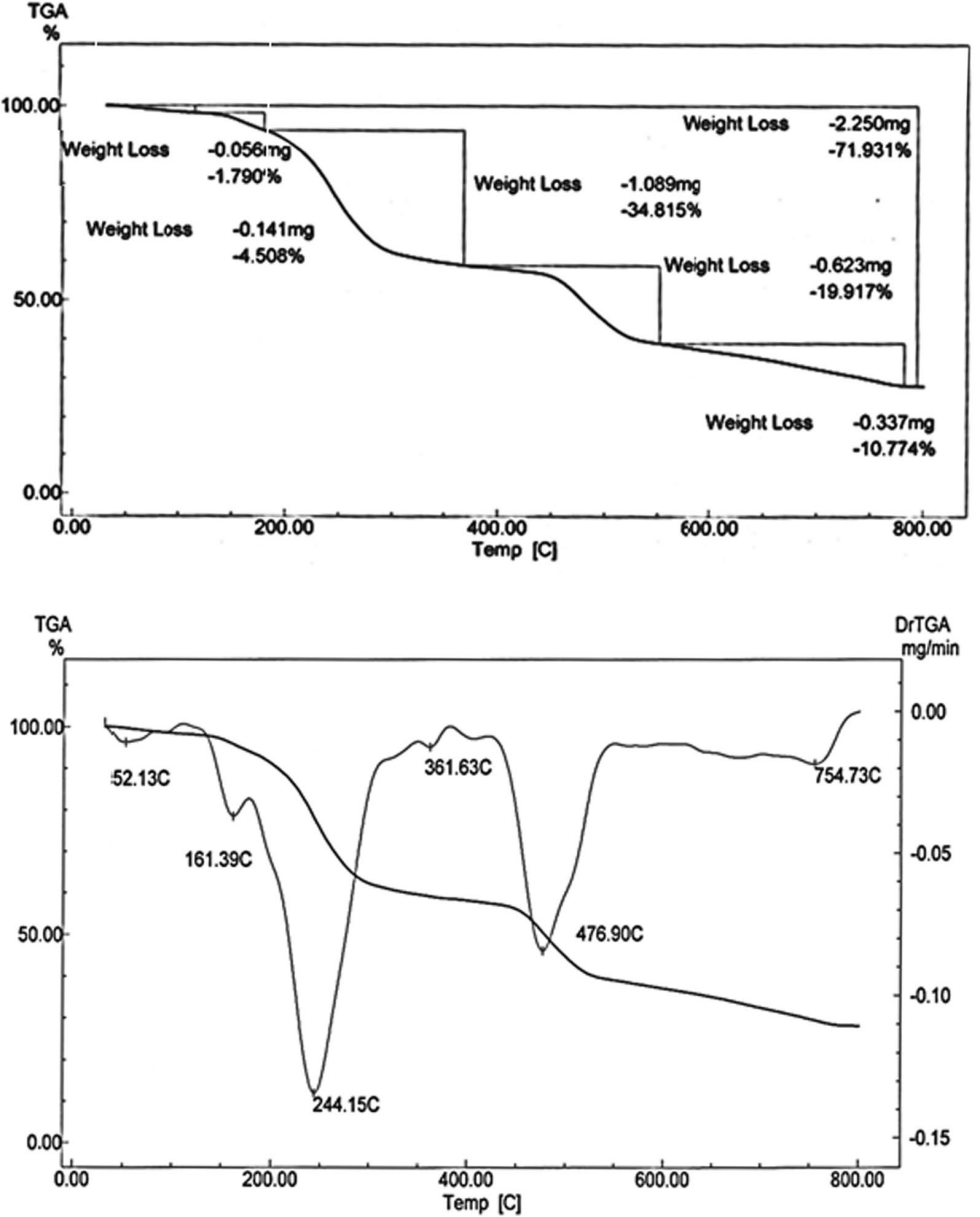
TGA curve of [Cu(L–H)Cl(EtOH)(H_2_O)].½EtOH.½ H_2_O **(1)**

**Fig. 5:**
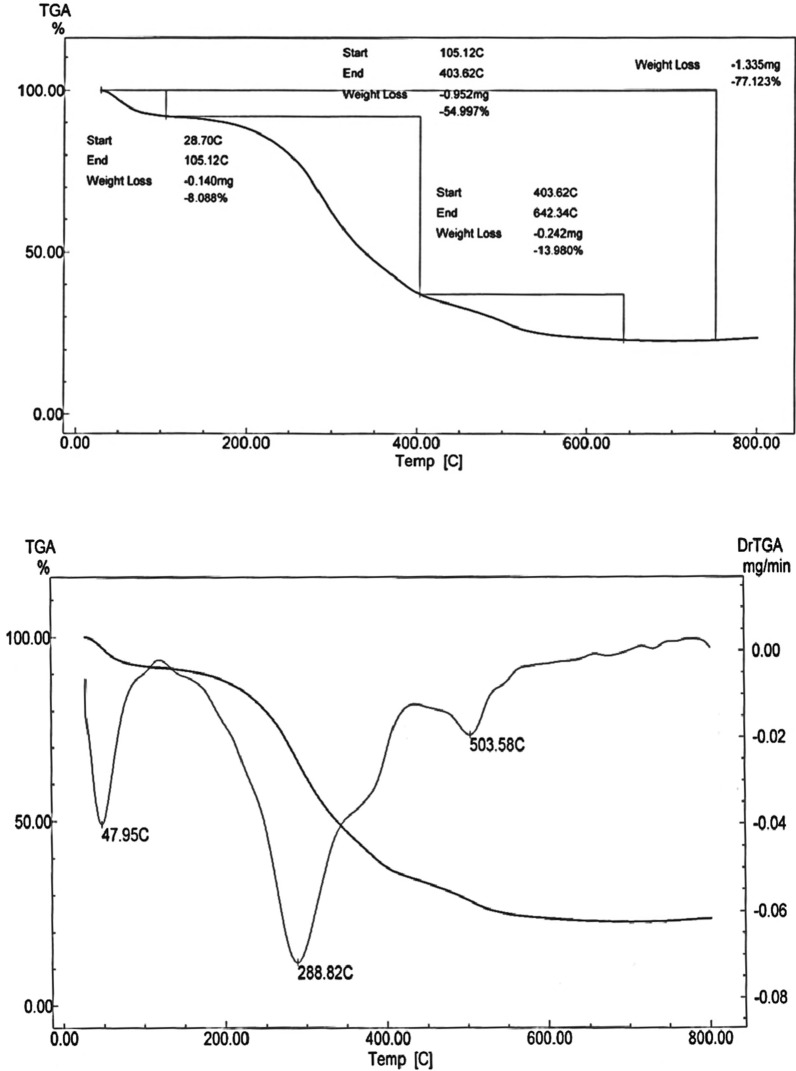
TGA curve of [Co(L-H)Ac(EtOH)(H_2_O)].EtOH(**2**)

**Fig. 6:**
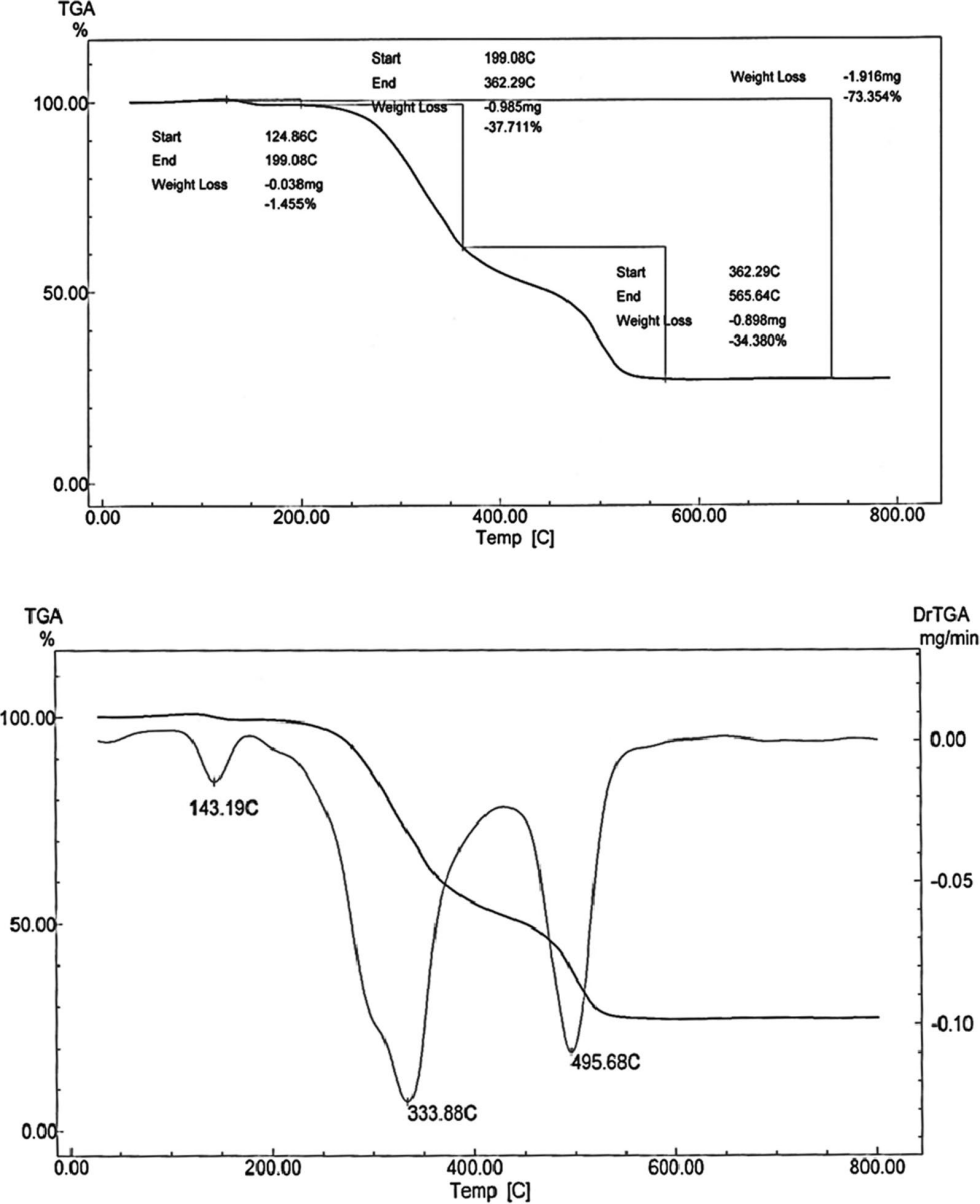
TGA curve of [Ni(L-H) EtOH]Cl**(3)**

Thermogravimetric analysis (TGA) of metal complexes is presented in Figs. 4, 5, 6 while the thermoanalytical results of the complexes are given in Table 5.

**Table 5:** Thermoanalytical results of Cu(II), Co(II) and Ni(II) complexes of the ligand (L)

Compound; NO	Temperature Range (°C)		Loss in weight found(calc.)^a^%		Decomposition product loss
			Mass loss%	Total mass loss%	
1) [Cu(LH)Cl(EtOH)(H_2_O)].^1^/_2_EtOH.^1^/_2_H_2_O	1st	30–185	6.4 (6.3)	72.6 (71.931)	Loss of 1/2H_2_O.1/2EtOH(Lattice)
	2nd	185–367	34.7 (34.8)		Loss of C_4_H_11_NCl
	3rd	367–556	20.3 (19.9)		Loss of C_2_HN_2_SO
	4th	556–782	11.2 (10.8)		Loss of HNCl leaving CuO + 6C
2) [Co(L–H)AC(EtOH)(H_2_O)].EtOH	1st	28–105	8.68 (8.088)	76.22 (77.123)	Loss of EtOH(Lattice)
	2nd	105–404	54.5 (54.997)		Loss of EtOH + H_2_O(Coord) + C_7_H_15_O_3_N_3_Cl
	3rd	404–643	13.6 (13.98)		Loss of C_2_H_2_N_3_,LeavingCo + C_5_H_4_
3) [Ni(L–H)EtOH]Cl	1st	125–363	39.82 (39.17)	74.72 (73.55)	Loss of EtOH(Coord) + C_3_H_9_NCl
	2nd	362–565	34.9 (34.38)		Loss of C_5_H_4_N_3_OS Leaving NiC_4_H_5_O_1/2_

The TGA thermogram of the Cu(II) complex **(1)**, [Cu(L–H) Cl(EtOH)(H_2_O)].½EtOH.½ H_2_O shows four stages of decomposition (Fig. 4). The first stage lies in the temperature range 30–185 ºC corresponding to the loss of the lattice water and ethanol molecules (6.3% close to the calculated value 6.4%). The second stage lies in the temperature range 185–367 ºC due to loss of the coordinated water, ethanol molecules and loss of C_4_H_11_NCl (found: 34.8%; calcd.: 34.7%). The third stage, which takes place between 367 and 556 °C, is attributed to the loss of C_2_HN_2_SO (19.9%, very close to the expected value of 20%). Loss of HNCl (observed: 10.8%; calculated: 11.2%) is due to the fourth stage, which takes place between 556 and 782 °C. The mass loss of 28.2% is close to the predicted value of 30.5% (the remaining residue is CuO + 6C). The total mass loss is 71.931% (the estimated value is 72.6%).

The TGA curve of the Co(II) complex, [Co (L–H)Ac(EtOH) (H_2_O)]. EtOH **(2),** shows three stages of decomposition as shown in Fig. 5. The first step lies in the 28–105 ºC range corresponds to the loss of ethanol molecules in lattice (found: 8.68%; calcd.: 8.08%). The second step of decomposition indicates the loss of the two coordinated ethanol, water molecules and C_5_H_12_ON_3_Cl (found: 54.997% close to the calculated 54.5%. This step lies in the 105–404 °C range.

The third stage in the 404–643 ºC range is attributed to the loss of C_2_H_2_NS (found: 13.98%; calcd.: 13.6%). The residual part of the complex is Co + C_5_H_4_. The observed mass loss is 22.8% close to the theoretically calculated 23.2%). The overall mass loss is 77.123% (calcd. 76.2%).

The thermal analysis curve of the Ni(II) complex, [Ni(L–H) EtOH]Cl **(3)**, shows two stages of decomposition (Fig. 6). The first step in the 125–362 ºC range corresponds to the loss of the coordinated ethanol molecules and C_3_H_9_NCl_2_ (found: 39.17%; calcd.: 39.82%). The second step is observed in the 362–565 °C range. This step is attributed to the loss of C_5_H_4_N_3_OS (observed: 34.38%, while the theoretical value is 34.9%). The residual part is the NiC_4_H_5_O_½_. And the observed mass loss is 26.6% close to the theoretically calculated 27.1%). The overall mass loss is 74.7% (calcd. 73.55%).

### Computational studies

#### Geometry optimization

**Fig. 7:**
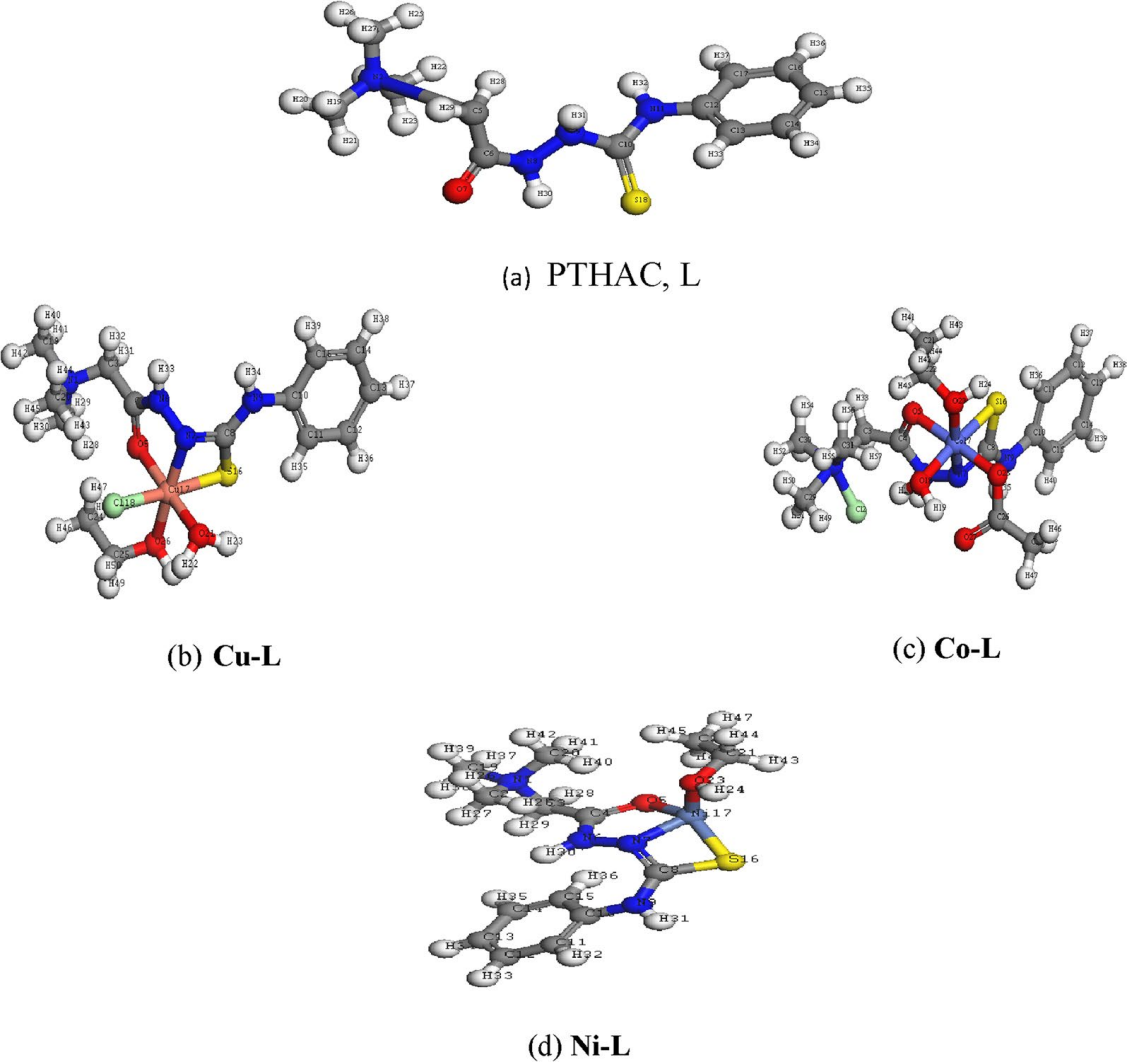
Optimized structures of **a** PTHAC, L, **b** Cu-L **c** Co-L and **d** Ni-L

The optimized geometries of the (PTHAC) ligand and its Co^2+^, Ni^2+^, and Cu^2+^complexes are shown in Fig. 7. Table 6 and (Additional file 1: Table S2–S9) display the bond lengths and angles of Co^2+^, Ni^2+^ and Cu^2+^complexes. For the Cu^2+^complex and Co^2+^ complex, metal ions are hexacoordinated in an octahedral geometry according to illustrating bond angle in Table 6. Meanwhile Ni.^2+^ complex tetracoordinated in a distorted tetrahedral geometry in which bond angles are illustrated in Table 6 [31, 32].

**Table 6:**
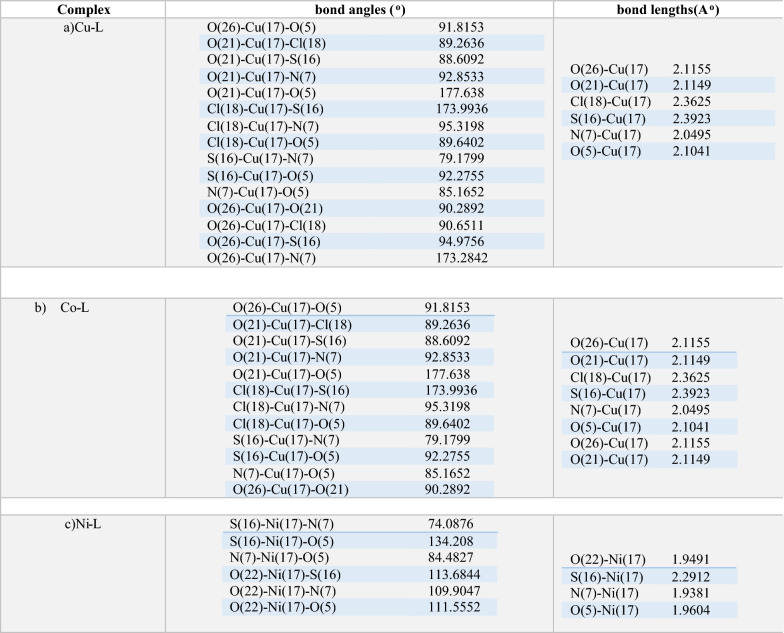
Selected bond lengths and angles of **Cu-L, Co-L** and Ni-L complexes

#### Global Chemical Reactivity Descriptors (GCRD)

**Fig. 8:**
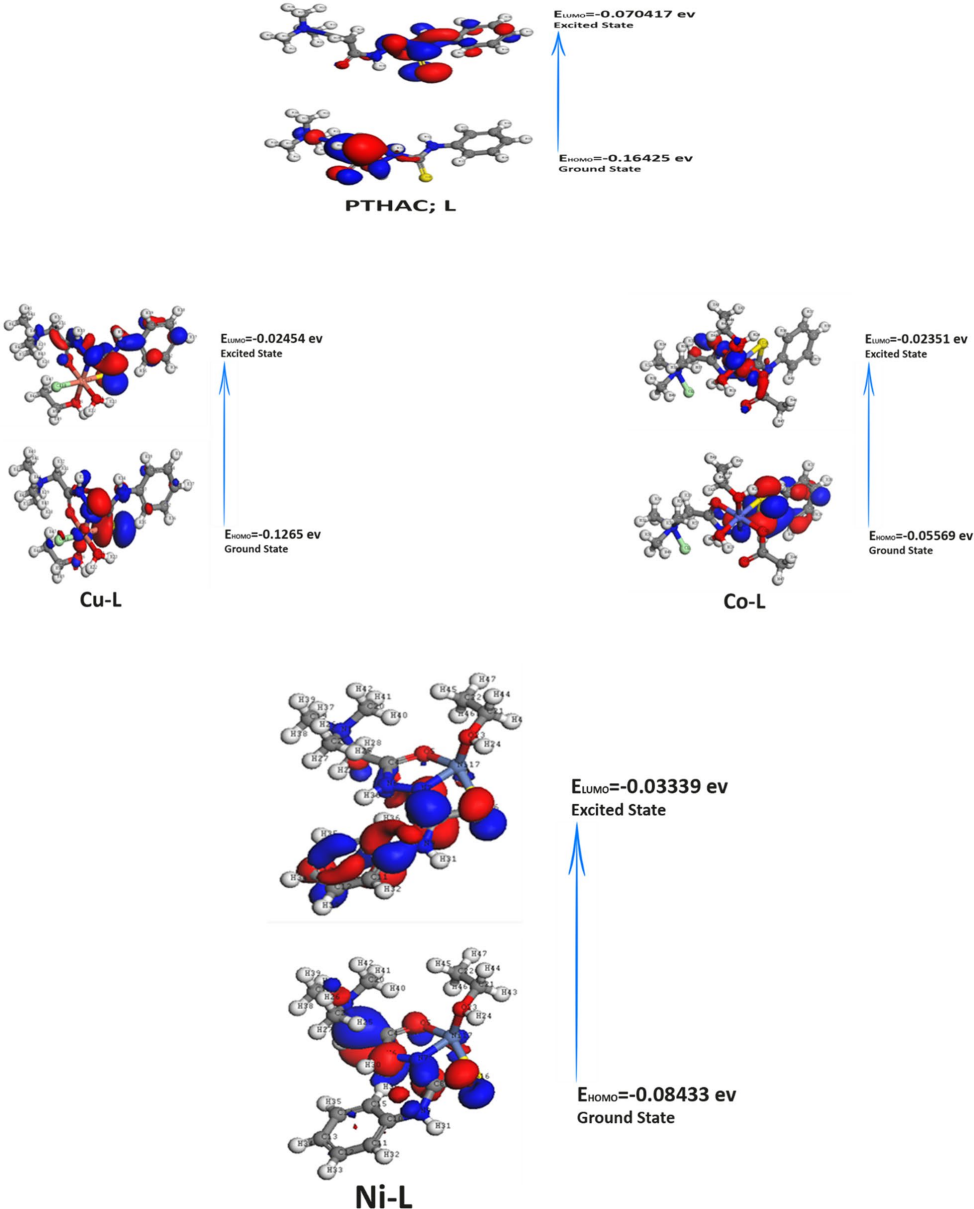
HOMO and LUMO energy level images of the PTHAC, L and its Co^2+^, Ni^2+^, and Cu^2+^complexes

The two most important components of theoretical molecular design are highest occupied molecular orbitals (HOMO) and lowest unoccupied molecular orbitals (LUMO). The HOMO–LUMO gap can be used to predict the molecular hardness and softness of a compound because the HOMO and LUMO sites are electron donor and acceptor sites, respectively. The HOMO and LUMO of optimized geometry of the PTHAC, L ligand and its Co^2+^, Ni^2+^, and Cu^2+^complexes are shown in Fig. 8. The Global Reactivity parameters such as ionization potential (IP), electron affinity (EA), hardness (η), softness (σ), electronegativity (χ), electrophilicity index (ω), and chemical potentials (µ)] can be determined from the HOMO and LUMO orbital energies through Koopman’s theorem. The energy gap (ΔE), electronegativity ($\chi$), hardness ($\eta$), chemical potentials (µ), softness ($\sigma$), and electrophilicity index (ω) were illustrated in Table 7. The energy gap values designate the reactivity of the compound for the metal surface (as the energy required for the transition from HOMO to LUMO increases, the reactivity decreases).

**Table 7:** The Global Chemical Reactivity Descriptors (GCRD)

Property	PTHAC,L	Cu-L	Co-L	Ni-L
E_HOMO_ [eV]	− 0.16425	− 0.1265	− 0.05569	− 0.08433
E_LUMO_ [eV]	− 0.070417	− 0.02454	− 0.02351	− 0.03339
ΔE_(LUMO–HOMO)_ [eV]	0.093833	0.10196	0.03218	0.05094
η [eV]	0.0469165	0.05098	0.01609	0.02547
σ [eV]^−1^	21.31446293	19.6155355	62.15040398	39.26187672
I_p_ [eV]	0.16425	0.1265	0.05569	0.08433
E_A_ [eV]	0.070417	0.02454	0.02351	0.03339
χ [eV]	0.1173335	0.07552	0.0396	0.05886
µ [eV]	− 0.1173335	− 0.07552	− 0.0396	− 0.05886
ω [eV]	0.146719707	0.055936352	0.048730889	0.068011378
Total energy (kcal mol^−1^)	− 728,969.9533	− 2,191,623.678	− 1,883,073.115	− 1,771,492.75
The dipole moment [Debye]	3.7766	14.9896	13.31989	17.1919

The ΔE _(LUMO–HOMO)_ for Cu-complex was found to be less reactive than that of the ligand **PTHAC, L.** On the other hand, Co(II), Ni(II) complexes were found to be more reactive than the ligand **PTHAC, L**

Soft molecules (σ) have a small energy gap compared to hard molecules (η), which have a larger energy gap. A soft molecule is more reactive than a hard molecule because a soft molecule has a lower ΔE (LUMO–HOMO). From Table 7, Co^2+^, Ni^2+^ complexes are softer than ligand **PTHAC, L** and this confirms that Co^2+^, Ni^2+^ complexes are more reactive than ligand **PTHAC, L**.

The electronegativity (χ) is a measure of power of atom(s) to attract the electrons. A high value of electronegativity (χ) suggests strong ability to attract electrons from the ligand, which leads to greater interaction to form the complex. The electronegativity (χ) decreases according to the following order:

PTHAC, L < Cu-L < Ni-L < Co-L

Chemical potential (μ), which estimates the escaping capability of electrons from the equilibrium framework, increases according to the following order.

PTHAC, L < Cu-L < Ni-L < Co-L

### Evaluation of the antitumor activity

**Fig. 9:**
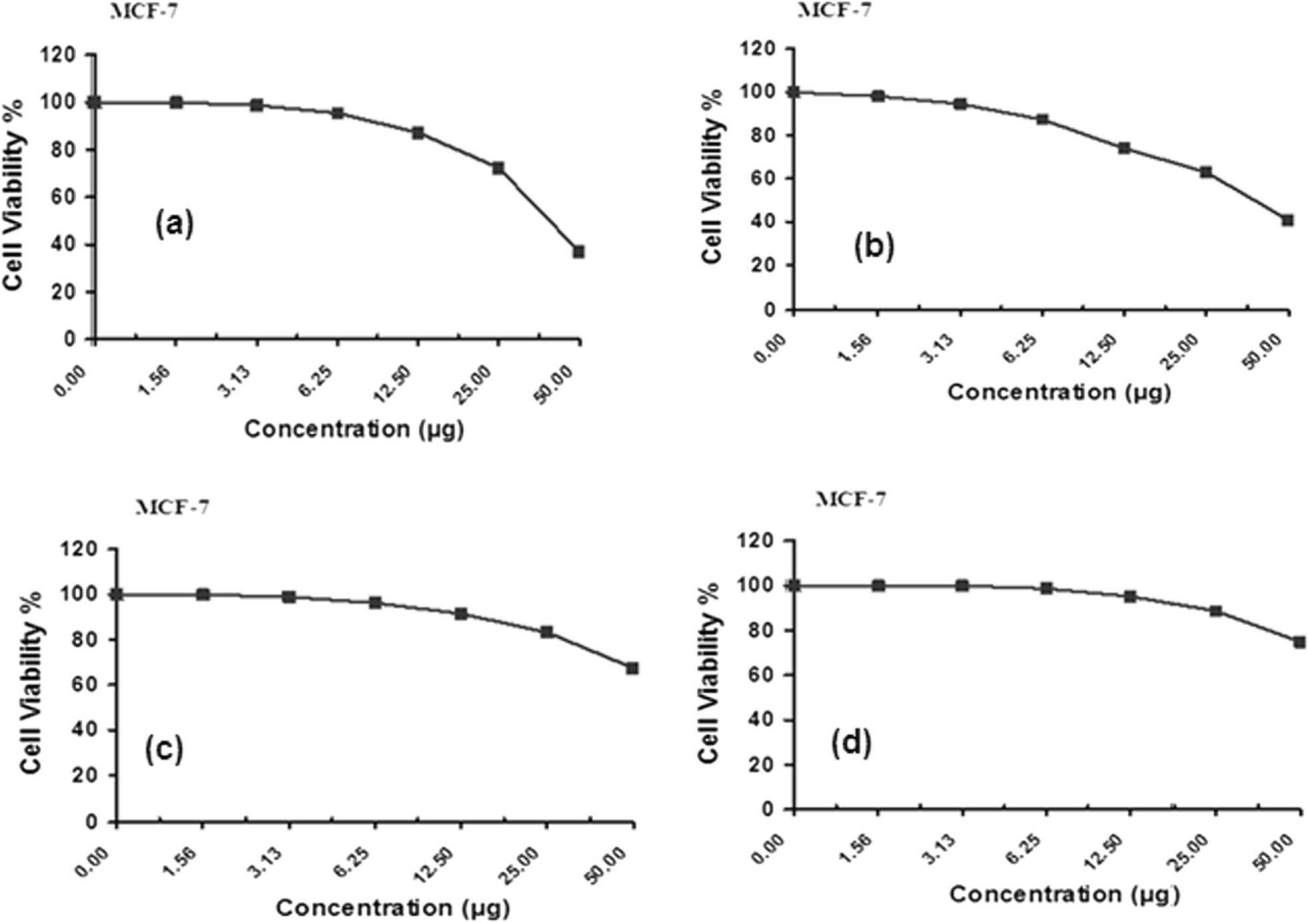
Antitumor activity of **a** the ligand (PTHAC; L), **b** [Cu (L-H)Cl(EtOH)(H_2_O)].½EtOH.½ H_2_O, **c** [Co (L-H)Ac(EtOH)(H_2_O)]EtOH and **d** [Ni(L-H)EtOH]Cl

**Fig. 10:**
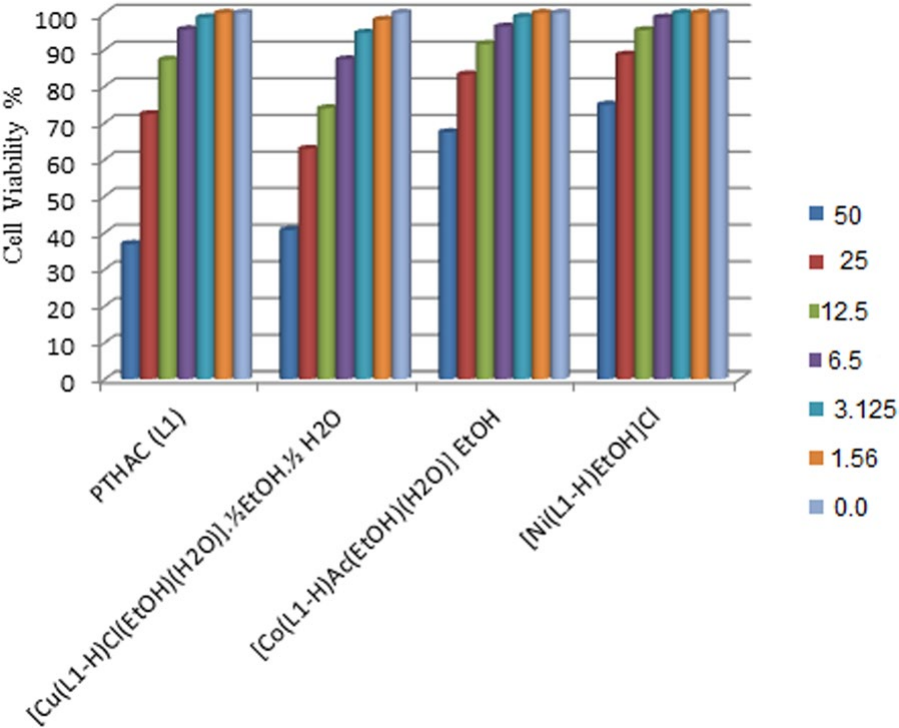
Antitumor activity of (PTHAC; L) and its metal complexes

The PTHAC ligand and its complexes(1–3) have been tested against breast carcinoma cells, Figs. 9 and 10 and the results are listed in Table 8.

**Table 8:** Antitumor activity of PTHAC and its metal complexes(1–3)

NO	Compound	Sample conc. (µg)	Viability %
(1)	PTHAC (L)	50	36.89
		25	72.36
		12.5	87.25
		6.25	95.49
		3.125	98.78
		1.56	100
		0	100.00
(2)	[Cu(L–H)Cl(EtOH)(H_2_O)].½EtOH.½ H_2_O	50	40.73
		25	62.89
		12.5	73.94
		6.25	87.32
		3.125	94.56
		1.56	98.14
		0	100.00
(3)	[Co(L–H)Ac(EtOH)(H_2_O)].EtOH	50	67.41
		25	83.22
		12.5	91.50
		6.25	96.27
		3.125	98.96
		1.56	100
		0	100.00
(4)	[Ni(L–H)EtOH] Cl	50	74.93
		25	88.62
		12.5	95.28
		6.25	98.71
		3.125	100
		1.56	100
		0	100.00

The IC_**50**_ values for the ligand and its complexes were compared with anticancer agent utilized at the present time. The results (Fig. 9a) indicate that the ligand (PTHAC; L) has an inhibitory activity against breast carcinoma cells with a value IC_50_ = *40.8 µg.* [Cu(L–H)Cl(EtOH)(H_2_O)].½EtOH.½H_2_O has an inhibitory activity against breast carcinoma cells under the same experimental conditions giving a value of IC_50 =_ 39.5 µg. As it is demonstrated in Fig. 10b, the cytotoxic activity of Cu-L is greater than that of the ligand (PTHAC). The other two complexes with the general formulae, [Co(L–H)Ac(EtOH)(H_2_O)].EtOH, [Ni(L–H)EtOH]Cl (Fig. 9c, d) give weak inhibitory activities against breast carcinoma cells under the same experimental conditions with values IC50 ≥ 50 µg.

Additionally, the IC_50_ of [Cu(L–H)Cl(EtOH)(H_2_O)].½EtOH.½H_2_O is comparable with Doxorubicin. The anticancer activity of [Cu(L–H) Cl(EtOH)(H_2_O)].½EtOH. ½H_2_O may be assigned to its attachment to cellular Fe pools. Consequently, the enzyme responsible for the conversion of ribonucleotides to deoxyribonucleotides, ribonucleotide reductase(RR), is rendered inactive. It is known that the RR activity is positively correlated with tumour cell proliferation. Deoxyribonucleotides are not created when RR activity is suppressed. Since these substances block DNA synthesis, they slow the proliferation of tumour cells and reduce cancer's overall population. Changes in the reductive conversion of ribonucleotides to deoxyribonucleotides seem to be responsible for the antitumor effect by inhibiting DNA synthesis in cancer cells [55].

### Molecular docking studies

**Fig. 11:**
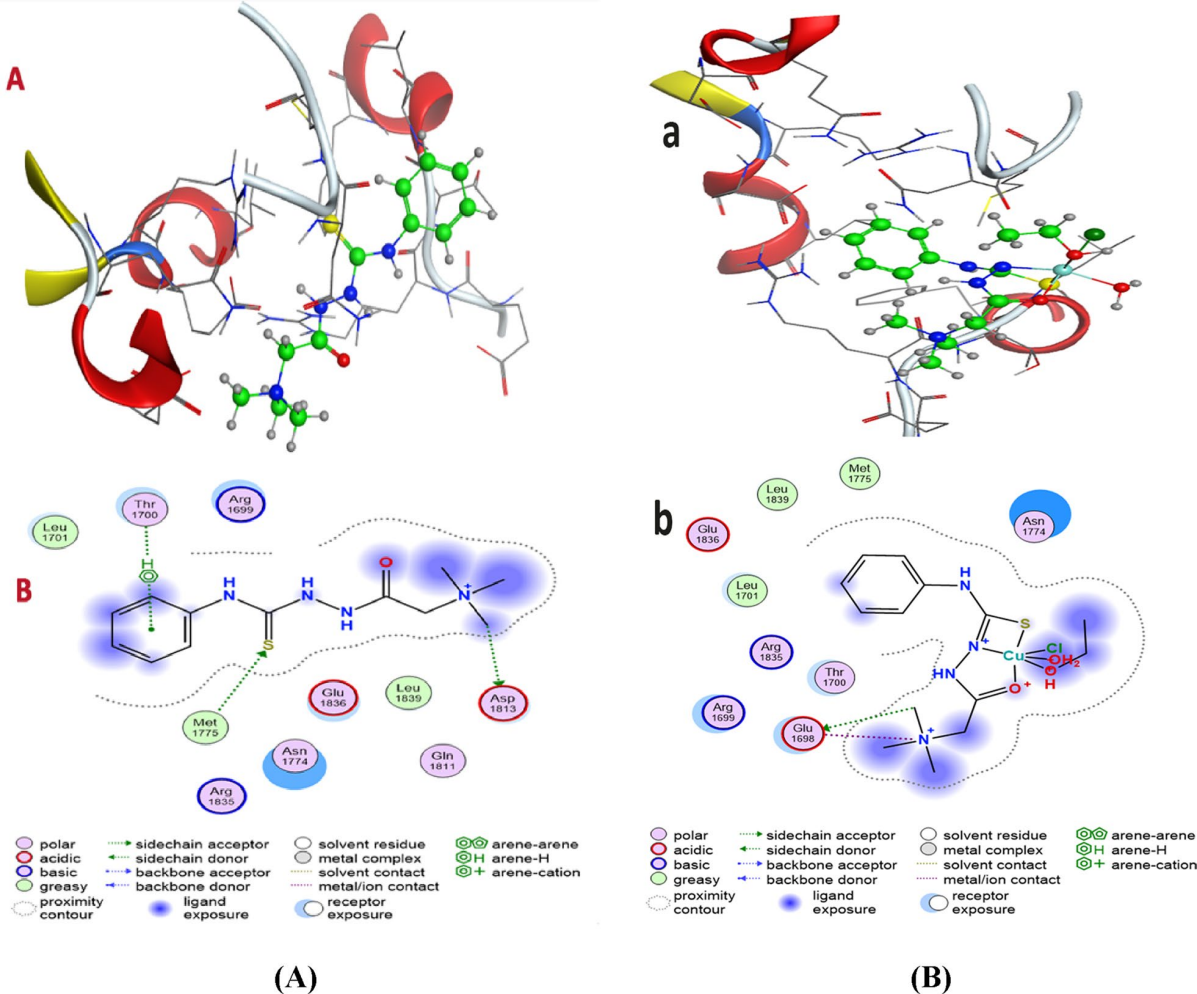

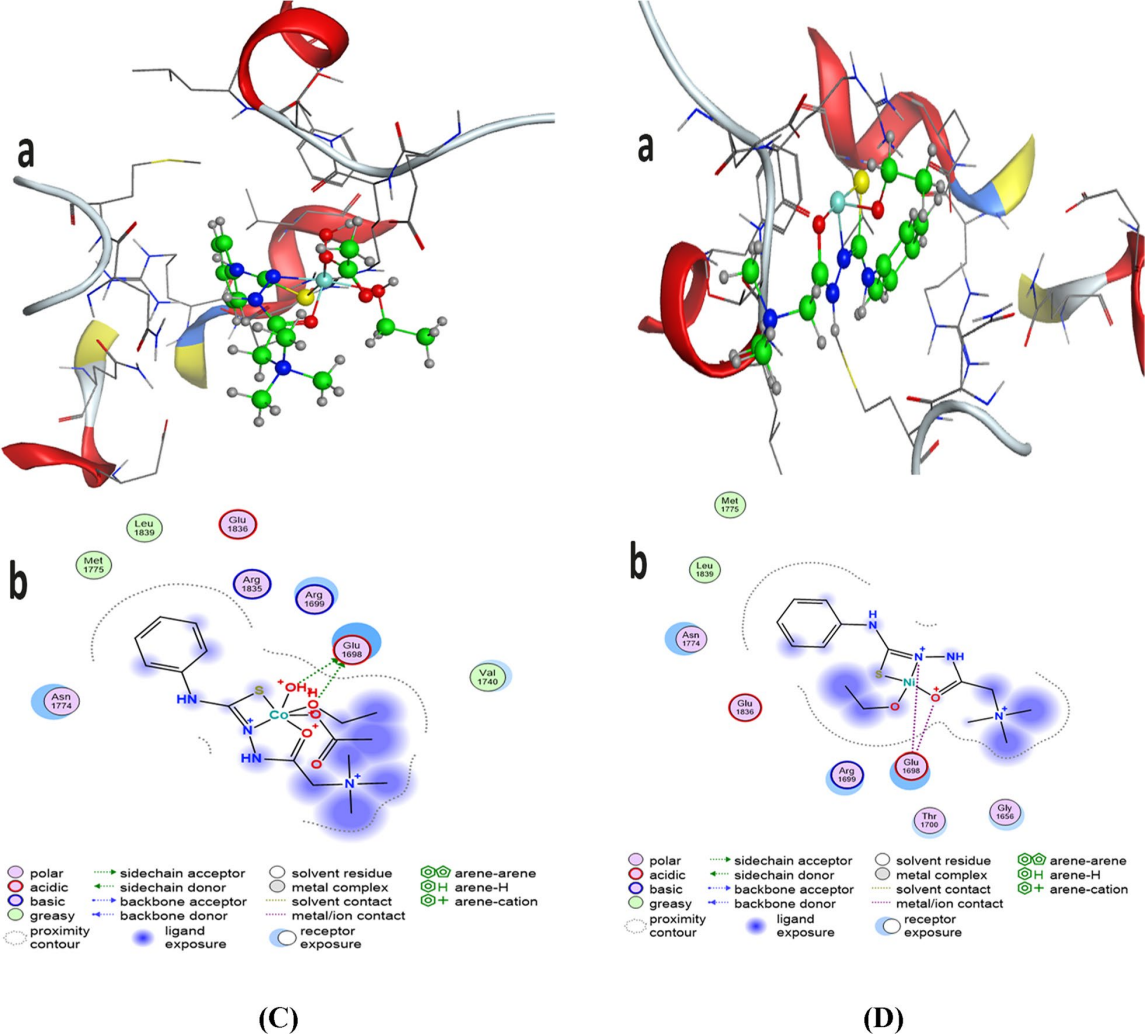
**a** 3D(a) and 2D(b) molecular interaction of **A** L & **B** Cu-L for inhibitor to breast cancer. **b** 3D(a) and 2D(b) molecular interaction of **C** Co-L & **D** Ni-L for inhibitor to breast cancer

Molecular docking studies were performed with breast cancer (PDB ID: 1jnx) to evaluate the preferred binding site of L and its complexes towards these targets (Fig. 11 a, b). From the docking data analysis (Table 9), the binding energies (best docking scores) in kcal/mol are a arranged as follow: Ligand (− 5.2629) > Cu-L (− 5.2256) > Co-L(− 5.1482) > Ni-L (− 5.1256) against breast carcinoma cells. This means that the ligand and its complexes have high ability to inhibit growth of breast carcinoma cells, which agree with experimental data. Meanwhile, Table 10 illustrates the Molecular docking interactions predicted for inhibitor binding with breast cancer for both L & its complexes [38, 56, 57].

**Table 9:** Molecular docking best docking scores (S) & rmsd_refine to breast cancer

Compound	Breast cancer	
	Best docking scores (S)	Rmsd_refine
L	− 5.2629	1.7505
Cu-L	− 5.2256	1.6491
Co-L	− 5.1482	1.849
Ni-L	− 5.1256	1.7701

**Table 10:** Molecular docking interactions predicted for inhibitor binding to breast cancer

Compound	Breast cancer		
	Hydrogen bonds	Stacking	Type
	Donor acceptor	interaction	
L	S → ASP A64	Benzene ring–Thr1700	Side chain, arene-H
	Met 1775 → S	–	Side chain,
	CH_3_ → ASP 1813	–	Side chain,
Cu-L	CH_3_ → Glu 1698	–	Side chain
	N^+^ → Glu 1698	–	Metal/ion contact
Co-L	H_2_O^+^ → Glu 1698	–	Side chain
	ETOH^+^ → Glu 1698	–	Side chain
Ni-L	O^+^ → Glu 1698	–	Metal/ion contact
	N^+^ → Glu 1698	–	Metal/ion contact

### Analytical studies separation via flotation and spectrophotometric determination of Co(II) using PTHAC

#### Influence of pH

**Fig. 12:**
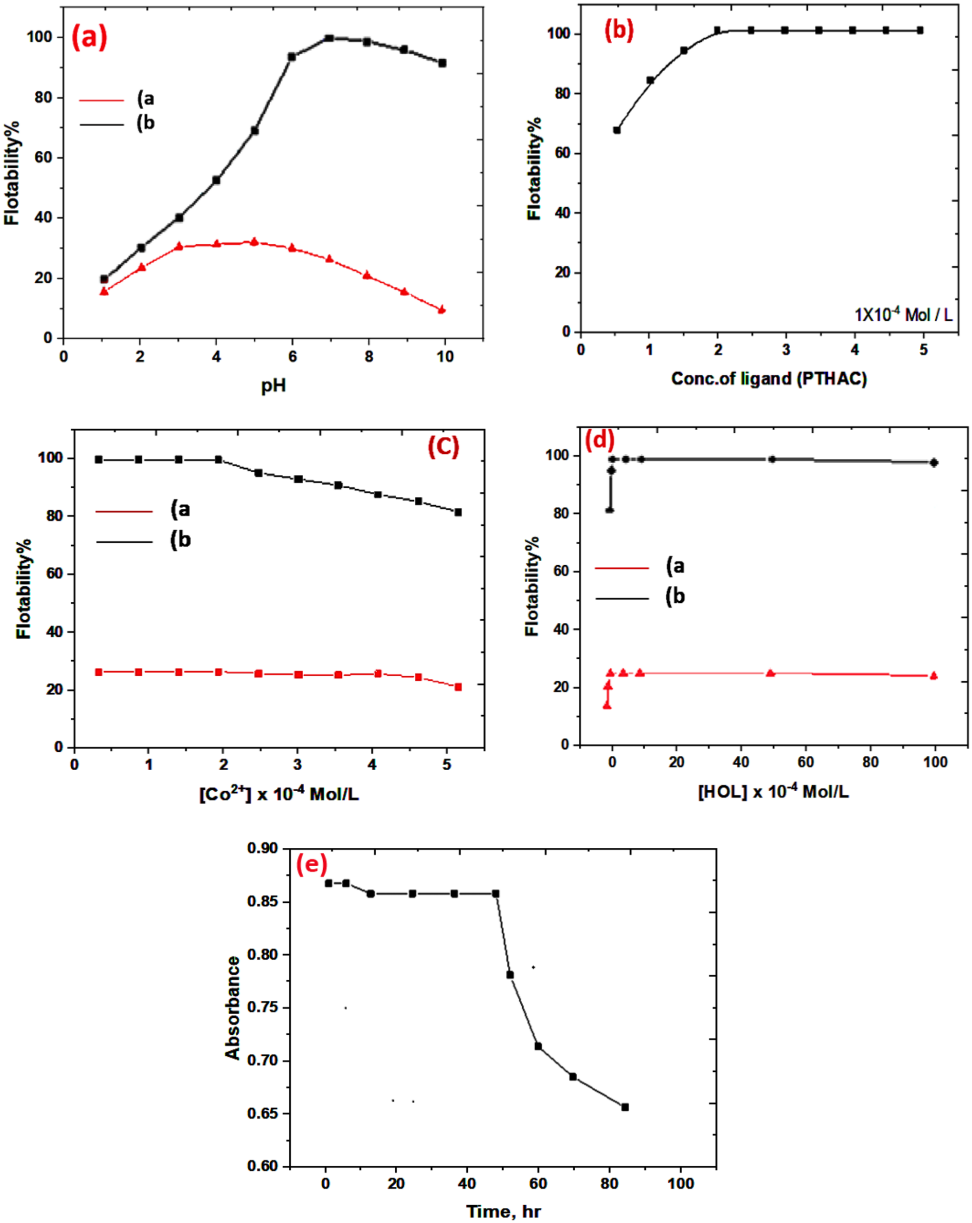
Effect of experimental variables on the flotation of Co(II), **a** Effect of pH; **b** Effect of PTHAC; **c** Effect of Co(II) concentrations; **d** Effect of HOL concentration; **e** Effect of time

The pH of the solution is essential for generating metal chelates and initiating the flotation process. The effect of pH on the flotation of 5 × 10^–5^ M of Co(II) with 1 × 10^−4^ M HOL was evaluated in the pH range 2.0—9.0 in the absence and in the presence of 1 × 10^−4^ M PTHAC. The data are graphically shown in Fig. 12a. Throughout the whole pH range studied, graph (a) demonstrates that in the absence of PTHAC, the flotation effectiveness of Co(II) ions is markedly reduced. On the other hand, in the presence of 1 × 10^–4^ M PTHAC, the flotation efficiency is increased to 100% (graph b). The increased flotation efficiency is attributed to the formation of hydrophobic Co(II)-PTHAC complex that was easily separated by the HOL surfactant. At higher pH values, the decrease in flotation efficiency may be attributed to the formation of a white emulsion and excessive foaming caused by sodium oleate.

#### Influence of ligand concentration

A trial was made to float Co(II) ions using HOL surfactant in the absence of PTHAC. The flotation efficiency was only 20–40%. The effect of varied concentrations of PTHAC on the flotation efficiency of 2.0 × 10^–4^ M of Co(II) using 1.0 × 10^–4^ M HOL was evaluated. The results are shown in Fig. 12b**.** The best flotation efficiency (equal to maximal absorbance) is obtained at a 1:1 (Co(II): PTHAC) ratio. The flotation efficiency increases with increasing PTHAC concentration. Since an excessive amount of ligand has no competition effect on the flotation process, the method may be used for analysis of different samples with unknown Co(II) concentrations. Consequently, it is allowed to use excess ligand while evaluating cobalt in its native non-identified components.

#### Influence of analyte concentration

Various quantities of Co(II) ions were floated in a solution containing 2 × 10^−4^ M PTHAC and 1 × 10^–4^ M HOL at the ideal pH in order to confirm the findings shown in Fig. 12b. According to Fig. 12c, the floatability reaches 100 percent at analyte concentration of 2.0 × 10^−4^ M, which corresponds to a molar ratio of 1:1 (Co(II): PTHAC) (Fig. 12c). With increasing analyte concentration, flotation becomes less efficient. This might be the result of the insufficient PTHAC required to bind all of the metal ions in the solution.

#### Influence HOL concentration

The influence HOL concentration on the flotation efficiency of 2X10^−4^ M Co(II) in the presence of 2X10^−4^ M PHTAC at pH 6.5 was investigated. The results, Fig. 12d**,** are demonstrate that maximal metal ion floatability is achieved throughout a broad concentration range of HOL(1X10^−4^ M- 0.5X10^−2^ M). The effectiveness of flotation dropped as HOL concentration increased. Surfactant molecules will aggregate into microscopic balls (micelles) with greater HOL concentrations. Micelles, which persist in solution while competing with the colligend molecule (the Co(-PHTAC complex), degrade the efficiency of separation. Furthermore, the bubble size is affected by the concentration of surfactant, with smaller bubbles occurring at higher surfactant concentrations. As a result, the foam is smoother. Throughout this study, HOL was used at a concentration of 1X10^−4^ M.

#### Stability of Co(II)-PTHAC complex

The PTHAC formed an olive green complex with Co(II) with maximum absorption at 620 nm. The absorbance of Co(II)-PTHAC complex was measured over different times to establish the long-term stability of the complex. Figure 12e demonstrates that the colour is formed instantaneously and remained constant for over 48 h.

#### Influence of temperature

The Influence of temperature on the flotation efficiency of 2X10^−4^ M Co(II) was investigated over a wide range of temperature using 1X10^−4^ M HOL and 2X10^−4^ M PTHCA at pH 6.5. The results obtained indicated that the flotation process is not affected by temperatures up to 60 °C; hence, (25 ± 2 °C) was employed for the following studies.

#### Influence of volume

Several studies were carried out to identify the ideal conditions for floating different quantities of Co(II) analyte in a constant volume (10 ml). It was observed that 2 × 10^‐5^ moll^‐1^ is the lowest concentration of the analyte that can be extracted quantitatively and safely from 10 ml. A second series of experiments was displayed to identify the ideal conditions for flotation of a constant concentration of the Co(II) (2 × 10^‐3^moll^‐1^)from a variety of aqueous volumes using adequately sized flotation cells. According to the findings, a preconcentration factor of 200 permits the separation of cobalt ions from aqueous volumes as small as 1 × 10^‐3^ moll^‐1^ to as big as 2L to 10 ml HOL quantitatively. After 2.5L, the capacity to float decreases approximately to 30%.

#### Influence foreign ions

The tendency of PTHAC to form complexes with many metal ions was studied by examining the effect of inorganic metal ions on Co(II) ion flotation under optimal conditions. These exotic ions were chosen for the experiment as they exist naturally in both fresh and salt water. Table 11 presents an overview of the acceptable amounts of each ion for a maximum error of ± 5% in flotation efficiency. The majority of experimentally tested foreign cations had no influence on the recovery of Co(II) ions and the impacts of the remaining foreign ions were regarded insignificant (~ 12%). All of these interferences disappeared when the concentration of PTHAC was raised to 1X10^−3^ moll^‐1^.

**Table 11:** Effect of different ions on the floatability of 1 × 10^–4^ moll^−1^ of Co(II) using 1 × 10^–3^ moll^−1^ PTHAC, 1 × 10^–4^ moll^−1^ HOL at pH 6.5

Added ion	Floatability, (%)
5 × 10^–5^ moll^−1^	
Mg^2+^	97
Ca^2+^	92.5
Al^3+^	97.6
Pb^2+^	94.9
Mn^2+^	98.5
Cr^3+^	98.5
Cd^2+^	95.6
Hg^2+^	91.5
Zn^2+^	96.6
EDTA	58
KSCN	91.8

#### Influence of ionic strength

Table 12 shows how effect of ionic strength of the salts on the flotation efficiency of 1.0 × 10^–4^ M Co(II) ions using 1.0 × 10^–4^ moll^‐1^ HOL in the presence of 1.0 × 10^–3^ moll^‐1^ PTHAC at the optimal pH. In most cases, the salts employed to alter the ionic composition of water seem identical to those found in the source water. Because of this, it is clear that the ionic strength of the medium hasn't had any noticeable impact on the flotation process or the detection of cobalt.

**Table 12:** Effect of ionic strength on the floatability of 1 × 10^–4^ moll^−1^ of Co(II) using 1 × 10^–3^ moll^−1^PTHAC and 1 × 10^–4^ moll^−1^ HOL

Salt	Concentration (moll^−1^)	Floatability (%)
NaCl	0.1	97
	0.01	97.6
	0.001	98.3
KCl	0.1	97.4
	0.01	98.3
	0.001	98.6
CaCl_2_	0.1	76.5
	0.05	80.6
	0.001	90.2

#### Analytical characteristics

Cobalt(II) forms green colored complex with ligand (PTHAC) that were completely separated using HOL surfactant. The intensity of color in the scum layer is gradually developed within 20 min and remains constant for ~ 48 h. It has been found that, the intensity of the color increases with increasing metal concentration. Such findings lead us to determine Cobalt (II) spectrophotometrically.

##### Absorption spectra

**Fig. 13:**
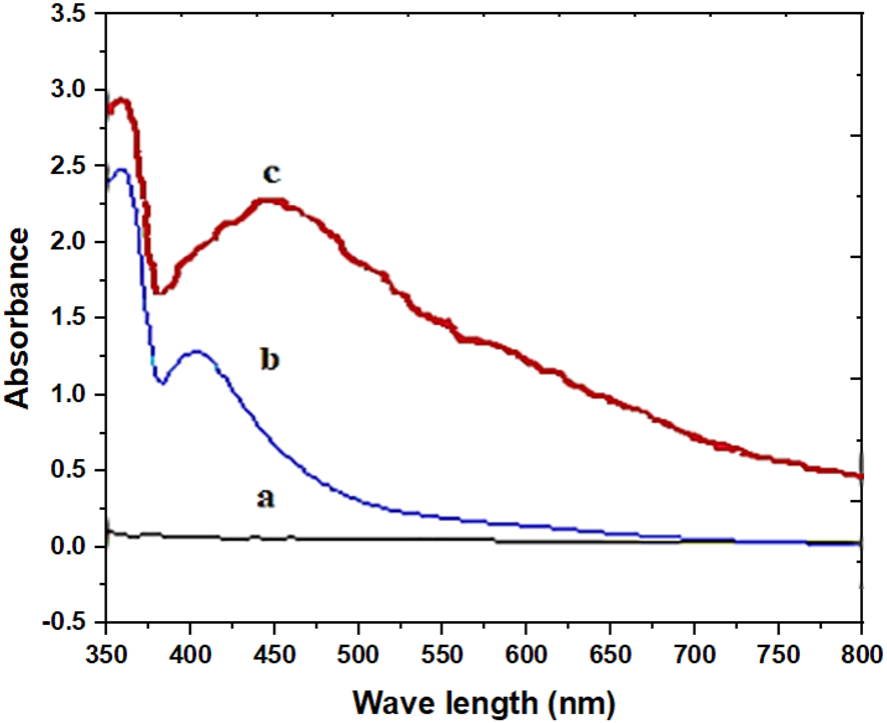
Absorption spectra of **a** 8 × 10^–4^ mol L^−1^ PTHAC, **b** Co-PTHAC complex, **c** Co – PTHAC -HOL complex

In both the aqueous and organic layers, Co-PTHAC exhibits absorption spectra that are distinct from that of PTHAC reagent. The peak absorbance of the PTHAC reagent is seen at 570 nm. Figure 13 represents the absorption spectra of the ligand PTHAC (Fig. 13a) and the Co-PTHAC complex in the aqueous phase and in the scum layer (Fig. 13b, c), respectively. Co-PTHAC exhibits maximal absorption at 620 nm (with a red shift of 50 nm), where the ligand has a negligible absorption at this wavelength. Furthermore, it was noticed that only one complex of Co-PTHAC is formed, and its extraction into the scum layer significantly increased the absorbance and, consequently, the sensitivity of the method, by comparing the absorption spectra of Co(II)-PTHAC in the aqueous (Fig. 13b) and in the scum layer (Fig. 13c).

Different concentration of cobalt (II) (1.66 × 10^–6^—2 × 10^–4^ moll^−1^; 0.1 -12 ppm) were added to a constant concentration of PTHAC (1 × 10^–3^ moll^−1^) and floated using 1 × 10^–4^ mol l^−1^ HOL. The absorbance values of the scum were measured at 620 nm. Beer's law is found to be applicable only at concentration up to 1.17 × 10^–4^ moll^−1^ (7 ppm). Above this range, the absorbance values did not vary linearly with the metal concentration i.e., the curve deviates from linearly. This deviation may be due to dissociation or association of the complexes in solution. The molar absorptivities are 0.14 × 10^4^ and 0.16 × 10^5^ l mol^−1^ cm^−1^ for the colored complex in the aqueous and scum phases, respectively. The analytical limit of detection of the standard aqueous solution is 0.04 mgl^−1^ Co(II), which corresponds to Sandell sensitivity of 3.7 × 10^–3^ µg.cm^−2^ and a relative standard deviation (n = 5) of 4.09%.

##### Mechanism of flotation

In flotation experiments, the role of surfactant is significant. To get closer to the true flotation process, it is necessary to investigate the nature of the interaction between the oleic acid surfactant and the prepared complex. The proposed procedure could work by producing a physical force, Van Der Waals contact, hydrogen bond formation between the hydrophilic portion of HOL and the active sites in the ligand complex, or coordination bond formation between oleic acid and the complex formed in solution may provide a species capable of self-floating (Analyte – Ligand – HOL).

**Fig. 14:**
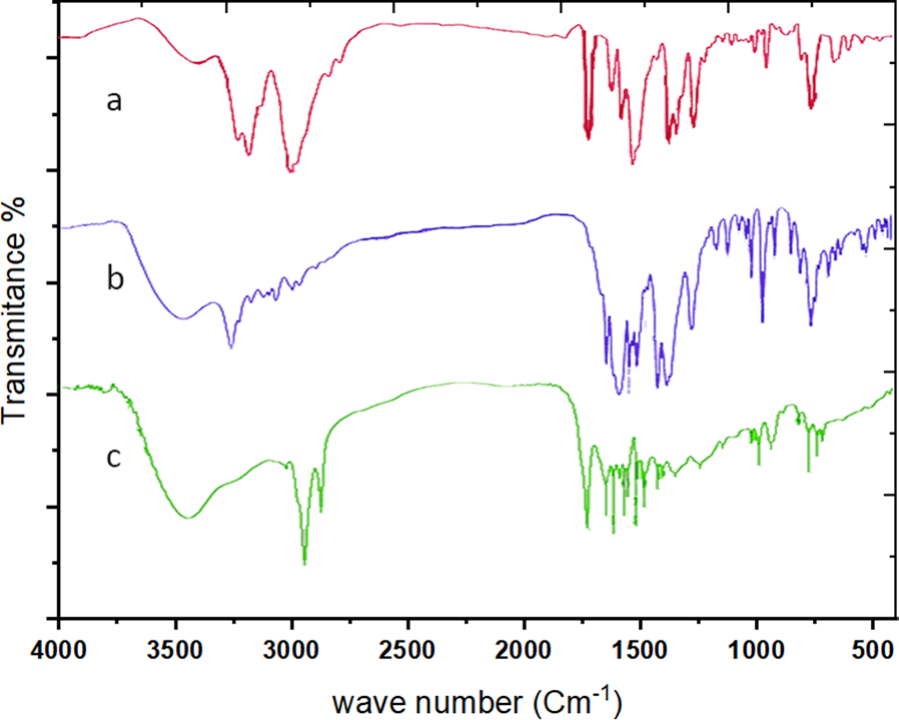
Infra-red spectra of **a** PTHAC, **b** Co-PTHAC complex and **c** Co – PTHAC – HOL floated complex

PTHAC interacts with cobalt(II) metal ions as a neutral tridentate ligand containing C = N, C = S, and C = O groups, as shown by its i.r. spectrum and the spectra of cobalt(II) complexes. The pH-dependent capacity of oleic acid to form hydrogen bonds with other systems allows it to exist in either its undissociated or dissociated form. The assignments of most major bands of PTHAC and its Co(II) complex are recorded in absence and presence of HOL Fig. 14 and Additional file 1: Fig. S6a–c.

The IR spectrum of PTHAC shows strong bands at 3239, 3191 and 3141 cm^−1^ which may be assigned to ʋ (NH)^1^, ʋ(NH)^2^ and ʋ(NH)^4^ vibrations, respectively. The ʋ (NH)^2^ band is disappeared wavenumbers in all the studied complexes, suggesting that the (NH)^2^ group is taking part in coordination.

The IR spectrum of Co-PTHAC complex shows disappearance of ʋ(C = S) band and shows other bands ʋ(C-S) at 677 cm^−1^, ʋ(C = N) at 1670 cm^−1^. Also, it shows the appearance of new bands in the low frequency region at ~ 524, 396 and 439 cm^−1^ due to ʋ(M–O), ʋ(M–N) and ʋ(M-S). Co (II) reacts with the PTHAC to form a green 1: 1 complex (**Structure IV**).

**Figure:**
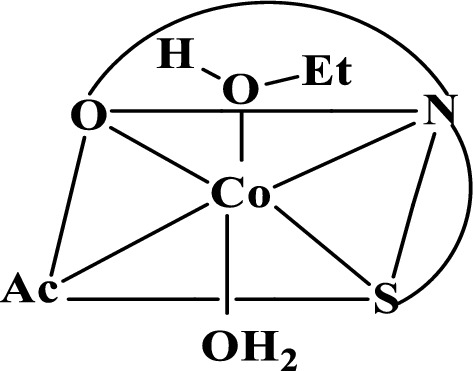
**Structure IV**

The IR spectrum of Cobalt (II)-PTHAC in HOL (Fig. 14c) after carefully washing many times with diethylether (to remove HOL traces of the surfactant) shows disappearance of ʋ(C = S) band and appearance of other bands ʋ(C-S) at 696 cm^−1^,ʋ(C = N) at 1629 cm^−1^. Also, the appearance of new bands in the low frequency region at ~ 550, 400 and 449 cm^−1^ due to ʋ(M–O), ʋ(M–N) and ʋ(M-S) and band at 1711 cm^−1^ may be owing to ʋ(C = O) vibration of the oleic acid carboxylate ion. One more band is also detected at ~ 1465 cm^−1^ assigned to ʋ_s_ COO^−^ vibration [58] of the oleic acid; the bands observed at 1957 and 1595 cm^−1^ are due to ʋ(O–H…O) and ʋ(O–H…N) vibration as the intramolecular hydrogen bonding.

**Scheme 5.**
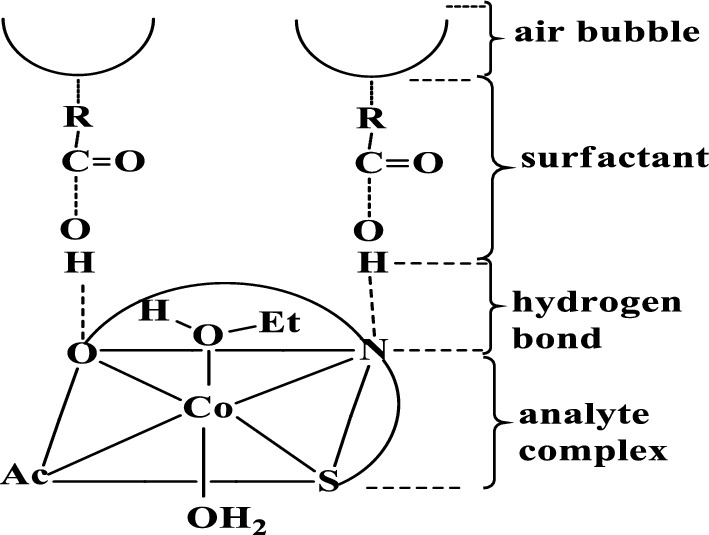
Mechanism of flotation of Co(II)

In consequence, as seen in Scheme 5, the Co-PTHAC-HOL system became hydrophobic and floated with air bubbles throughout the flotation process [23].

##### Applications

###### Analysis of water samples

The recoveries of particular quantities of Co(II) added to real tap, river and sea water samples were analysed to determine the applicability of the proposed method to natural water samples (obtained from various locations). Five, ten, or fifteen mgl^−1^ of Co(II) were added to 30 ml aliquots of clean, uncontaminated, filtered water samples, and the pH was adjusted to less than five. Co(II) content was estimated spectrophotometrically and validated by FAAS after flotation. The findings are shown in Table 13.

**Table 13:** Determination of Co(II) spiked in natural water samples after flotation using 1.0 × 10^–2^ moll^‐1^ PTHAC and 1.0 × 10^–4^ moll^‐1^ HOL at pH ~ 6.5

Type of water (location)	Co(II) added	Recovery (%)	
	(mg l^−1^)	FAAS	Spectrophoto
Distilled water	5	99.6	100
	10	99	98.5
	15	100	100
Tap water	0	0	0
(Our laboratory)	5	99	99
	10	100	100
	15	99.7	100
Nile water	0	0	0
(Mansoura City)	5	100	100
	10	100	100
	15	99.5	99
Sea water	0	0	0
(Demiate City)	5	99.4	99.6
	10	99.7	100
	15	100	100
	0	0	0
(Ras El-Barr)	5	99.9	100
	10	99.6	99.4
	15	100	100

###### Analysis of ore samples

Analysis of lead–Zinc sulfide and steel scrap sample real ore samples was carried out in order to validate the proposed flotation procedure. The Co(II) concentration was identified in the mother liquor by FAAS with a recovery % of 100% and a relative standard deviation(RSD) ≤ 3% as shown in Table 14.

**Table 14:** Analysis of Co(II) at pH 6.5 in some ore samples and pharmaceutical samples in presence of 1 × 10^–2^ moll^−1^ PTHAC using 1 × 10^–4^ moll^−1^ HOL

Sample name	Analyte, ppm Co(II)	Found	Recovery	Abs. error	R. error	SD	RSD
	Certificate		%				
Lead–Zinc sulfide ore	1	1	100	0	0	0.017	1.7
Steel scrap sample	0.8	0.81	100.1	0.01	0.013	0.024ي	3
Biovet ampoule	44.5	44.4	99.8	0.002	0.001	0.019	1.07

## Pharmaceutical samples

The suggested preconcentration method was also applied to a pharmaceutical sample vitamin B_12_ (Biovet ampoule) that was dissolved in concentrated nitric acid and heated to near dryness. Then, the residue was diluted with hot doubly distilled water(DDW). The aforementioned steps of flotation were carried out. The Co(II) in the pharmaceutical sample was determined by FAAS. The results given in Table 14 denote that Co(II) ions could successfully be determined in pharmaceutical sample with recovery % of 99.8% and a relative standard deviation(RSD) < 2%.

## Conclusion

**Fig. 15:**
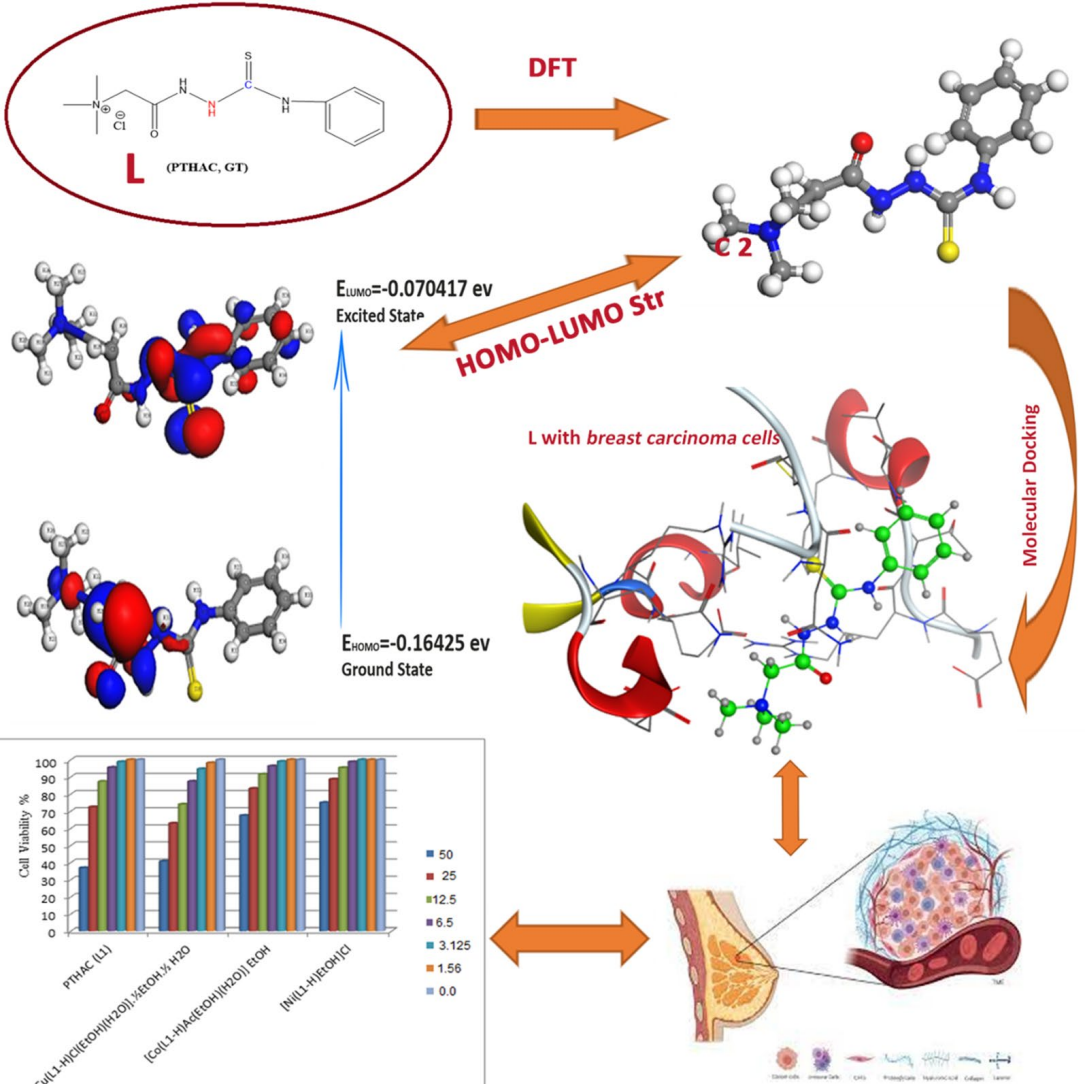
Schematic representation of the DFT, the molecular docking and the cytotoxic activity of PTHAC and its complexes

In the present study, phenyl isothiocyanate Girard-T (PTHAC) and its metal complexes with Cu(II), Co(II) and Ni(II) were prepared and characterized by elemental analyses, magnetic moment, spectra (IR, UV–Vis, ^1^H NMR, mass) and thermal studies. The chelation behavior of the ligand N-{[(phenylamino) thioxomethyl] hydrazino carbonyl methyl}trimethyl ammonium chloride (PTHAC) towards Cu(II), Co(II) and Ni(II) ions has been studied. Spectroscopic data showed that PTHAC acts as an ONS tridentate donor that forms a mononuclear complex with Cu(II), Co(II), and Ni(II) ions. The complexes were given an octahedral shape. The TGA and DTA were applied to obtain how stable the ligand and metal complexes were when heated. The lengths and angles of the bonds, the HOMO, LUMO, dipole moment, and charges on the atoms have been figured out. The cytotoxic activities of the PTHAC and the formed complexes against breast carcinoma cells have been investigated. Breast carcinoma cells are disrupted from developing more by the CuII-L complex than by the free ligand, the CoII-L, or the NiII-L. The ligand PTHAC was effectively used for the flotation and spectrophotometric measurement of Co(II) in a variety of media. Flotation of PTHAC-Co(II) complex was proposed to be due to hydrogen bond formation between the PTHAC-Co(II) complex and the HOL surfactant. The DFT, the molecular docking and the cytotoxic activity of PTHAC and its complexes are represented in Fig. 15.

### Supplementary Information

**Additional file 1:** **Fig. S1. **IR spectrum of ligand (PTHAC). **Fig. S2.** IR spectrum of [Cu(L^1^-H)Cl(EtOH)(H_2_O)].½EtOH.½ H_2_O**(1)**. **Fig. S3.** IR spectrum of [Co(L-H)Ac(EtOH)(H2O)].EtOH (2). **Fig. S4.** IR spectrum of [Ni(L-H)EtOH]Cl**(3)**. **Fig. S5.** **a** Mass spectrum of [Cu(L-H)Cl(EtOH)(H_2_O)].½EtOH.½ H_2_O (B). **b** Mass spectrum of [Co(L-H)Ac(EtOH)(H_2_O)].EtOH **(C).** **c **Mass spectrum of [Ni(L-H) EtOH] Cl**(D)**. **Fig. S6.** Infra-red spectra of **a** PTHAC, **b** Co- PTHAC complex and **c** Co–PTHAC–HOL floated complex. Additional tables.

## Data Availability

All data generated or analysed during this study are included in this published article.
