# Supplementary material for: Design, spectral, molecular modeling, antimitotic, analytical and mechanism studies of phenyl isothiocyanate Girard's T derived metal complexes
Source: BMC Chem. 2023 Nov 12;17(1):153. doi: 10.1186/s13065-023-01033-x (PMC10642003; doi:10.1186/s13065-023-01033-x)
Supplement: Supplementary file 1 — Additional file 1: Fig. S1. IR spectrum of ligand (PTHAC). Fig. S2. IR spectrum of [Cu(L1-H)Cl(EtOH)(H2O)].½EtOH.½ H2O(1). Fig. S3. IR spectrum of [Co(L-H)Ac(EtOH)(H2O)].EtOH (2). Fig. S4. IR spectrum of [Ni(L-H)EtOH]Cl(3). Fig. S5. a Mass spectrum of [Cu(L-H)Cl(EtOH)(H2O)].½EtOH.½ H2O (B). b Mass spectrum of [Co(L-H)Ac(EtOH)(H2O)].EtOH (C). c Mass spectrum of [Ni(L-H) EtOH] Cl(D). Fig. S6. Infra-red spectra of a PTHAC, b Co- PTHAC complex and c Co–PTHAC–HOL floated complex. Additional tables. [file 13065_2023_1033_MOESM1_ESM.docx]

**Additional file 1**

**Design, spectral, molecular modeling, antimitotic, analytical and mechanism studies of Phenyl isothiocyanate Girard's T derived metal complexes**

**Magda A. Akl^*1^, Nora A El Mahdy^1^, Zizi Elbadrawy^2^, Abdelrahman S. El-Zeny ^1^ and Mohsen M. Mostafa^1^**

**^1^** Chemistry Department, Faculty of Science, Mansoura, University, Mansoura 35516, Egypt

^2^Chemistry Department, Faculty of Science, Jizan University, Jizan, Kingdom of Saudi Arabia

***Corresponding author:**

Prof Magda A. Akl, Chemistry Department, Faculty of Science, Mansoura University, Mansoura, Egypt, E-mail: [magdaakl@yahoo.com](mailto:magdaakl@yahoo.com)

**
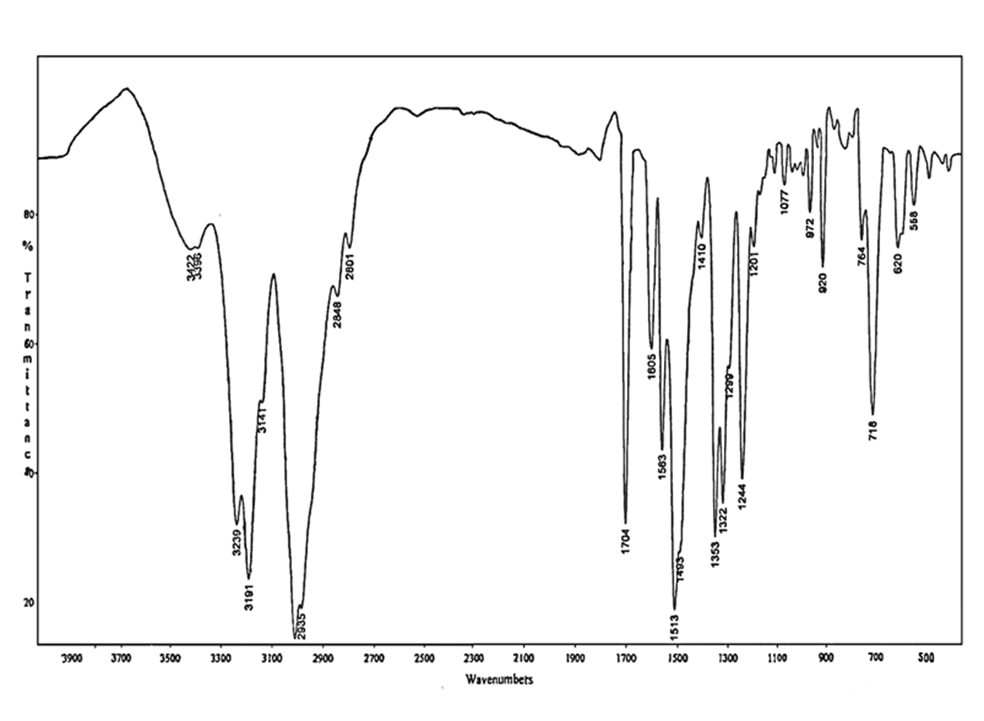
**

**Fig. S1.**  IR spectrum of ligand (PTHAC)


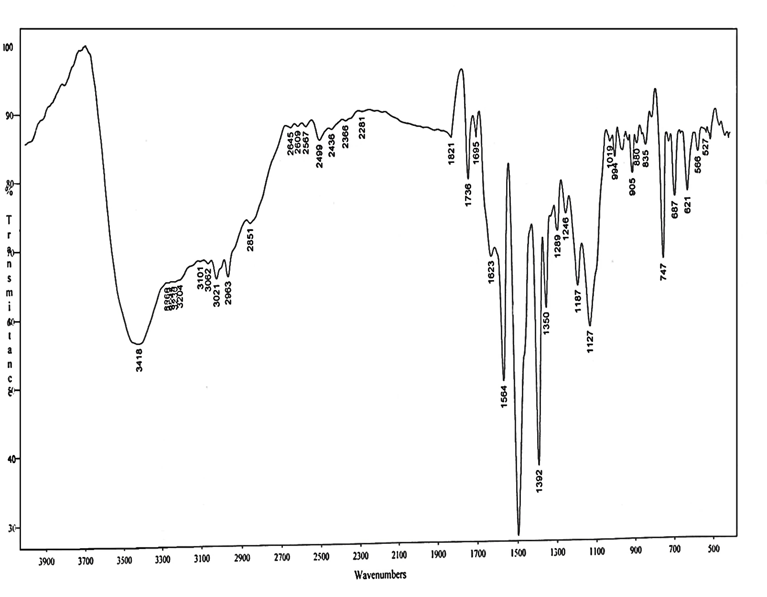
**Fig.S2.** IR spectrum of [Cu(L^1^-H)Cl(EtOH)(H_2_O)].½EtOH.½ H_2_O**(1)**.


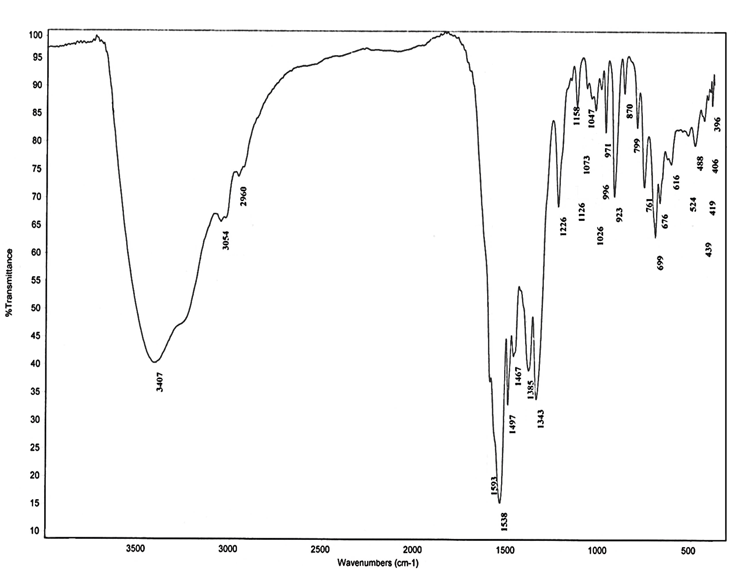


Fig.S3. IR spectrum of [Co(L-H)Ac(EtOH)(H2O)].EtOH (2).


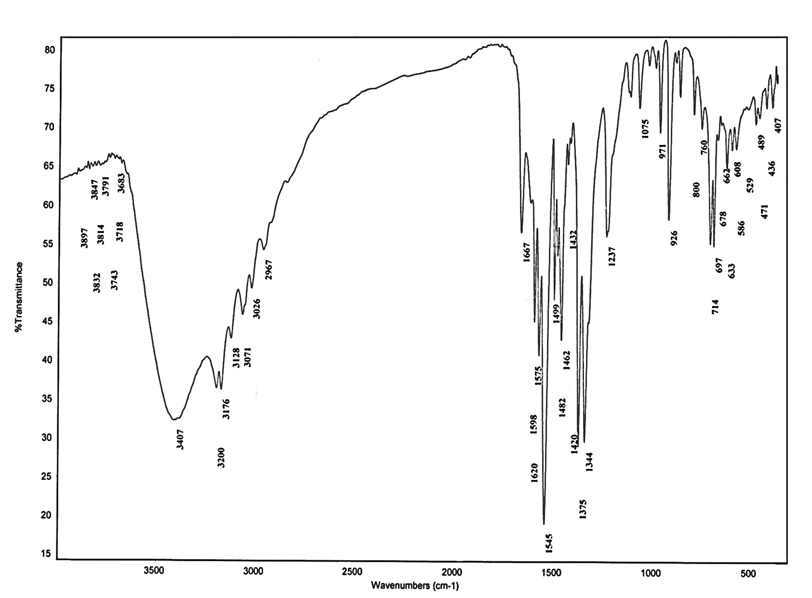


**Fig.S4.** IR spectrum of [Ni(L-H)EtOH]Cl **(3)**.


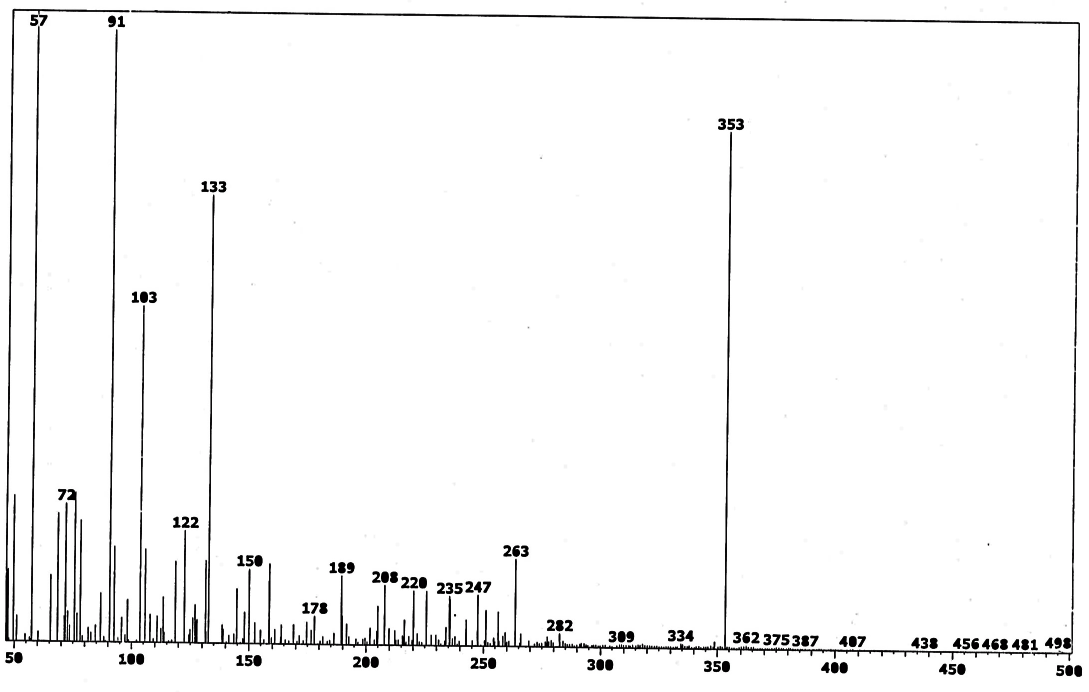


**Fig.S5a.** Mass spectrum of [Cu(L-H)Cl(EtOH)(H_2_O)].½EtOH.½ H_2_O (B).


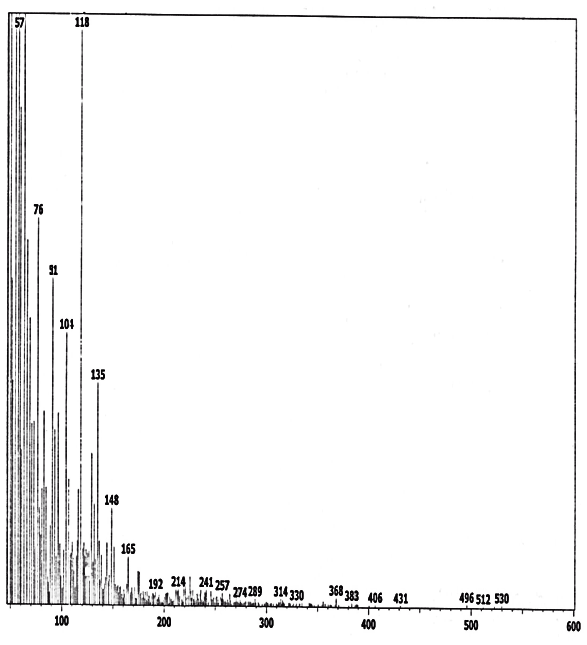


**Fig.S5b.** Mass spectrum of [Co(L-H)Ac(EtOH)(H_2_O)].EtOH **(C).**


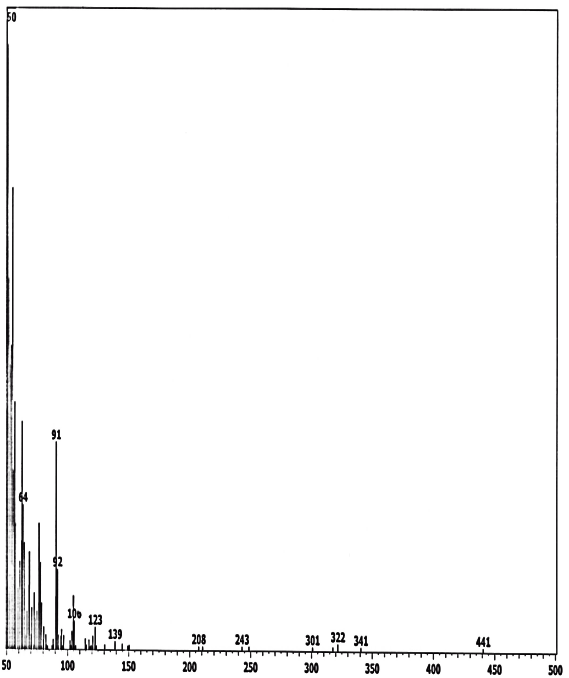


**Fig.S5c.** Mass spectrum of [Ni(L-H) EtOH] Cl **(D)**.


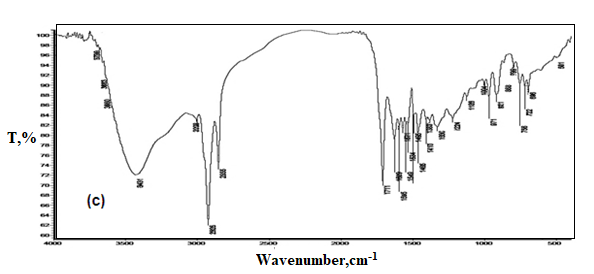


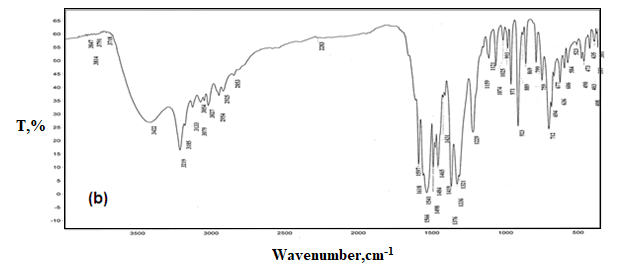


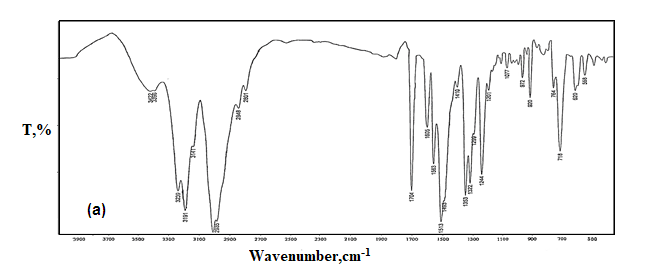


**Fig.S6.** Infra-red spectra of (a) PTHAC, (b) Co- PTHAC complex and (c) Co – PTHAC – HOL floated complex. The measurements were carried out in KBr discs .

**Table (1S)**: Solubility of the ligand (L^1^;PTHAC) and its metal complexes in water and some organic solvents.

| **Solvent** | **L** | **(1)** | **(2)** | **(3)** |
| --- | --- | --- | --- | --- |
| **Dist.H_2_O** | S | S | S | Ins. |
| **EtOH** | S | Ins. | Ins. | Ins. |
| **MtOH** | S | Ins.. | Ins. | Ins. |
| **Acetone** | S | S | Ins. | Ins. |
| **Acetonitril** | S | S | P.S. | Ins. |
| **CHCl_3_** | S | Ins. | Ins. | Ins. |
| **THF** | S | Ins. | Ins. | Ins. |
| **DMF** | S | S | P.S. | P.S. |
| **DMSO** | S | S | S | P.S. |

S= soluble P.S. = partially soluble Ins. = insoluble

**Table (2s)**: Bonds lengths (Å) of PTHAC using DFT-method from DMOL^3^calculations

| **Bond** | **Length(Å)** | **Bond** | **Length(Å)** | **Bond** | **Length(Å)** | **Bond** | **Length(Å)** |
| --- | --- | --- | --- | --- | --- | --- | --- |
| C(17)-H(37) | 1.0982 | C(12)-C(13) | 1.4319 | C(6)-O(7) | 1.2947 | N(2)-C(5) | 3.3242 |
| C(16)-H(36) | 1.0976 | N(11)-H(32) | 1.0345 | C(5)-H(29) | 1.0933 | N(2)-C(4) | 1.4602 |
| C(16)-C(17) | 1.4171 | N(11)-C(12) | 1.4038 | C(5)-H(28) | 1.0949 | N(2)-C(3) | 1.4589 |
| C(15)-H(35) | 1.097 | C(10)-S(18) | 1.7506 | C(5)-C(6) | 1.4175 | C(1)-H(21) | 1.1069 |
| C(15)-C(16) | 1.4227 | C(10)-N(11) | 1.4053 | C(4)-H(27) | 1.1084 | C(1)-H(20) | 1.1178 |
| C(14)-H(34) | 1.0968 | N(9)-H(31) | 1.0464 | C(4)-H(26) | 1.1162 | C(1)-H(19) | 1.1074 |
| C(14)-C(15) | 1.4209 | N(9)-C(10) | 1.4128 | C(4)-H(25) | 1.1084 | C(1)-N(2) | 1.4581 |
| C(13)-H(33) | 1.0947 | N(8)-H(30) | 1.0348 | C(3)-H(24) | 1.1179 |  |  |
| C(13)-C(14) | 1.4169 | N(8)-N(9) | 1.417 | C(3)-H(23) | 1.1041 |  |  |
| C(17)-C(12) | 1.4304 | C(6)-N(8) | 1.4257 | C(3)-H(22) | 1.1066 |  |  |

**Table (S3)**: Bonds angles (°) of PTHAC using DFT-method from DMOL^3^ calculations

| **Angle** | **Degree(°)** | **Angle** | **Degree(°)** | **Angle** | **Degree(°)** |
| --- | --- | --- | --- | --- | --- |
| H(37)-C(17)-C(16) | 120.4233 | S(18)-C(10)-N(11) | 124.0776 | H(26)-C(4)-H(25) | 108.2047 |
| H(37)-C(17)-C(12) | 119.6593 | S(18)-C(10)-N(9) | 123.6513 | H(26)-C(4)-N(2) | 112.0578 |
| C(16)-C(17)-C(12) | 119.8951 | N(11)-C(10)-N(9) | 112.2518 | H(25)-C(4)-N(2) | 109.2613 |
| H(36)-C(16)-C(17) | 119.7033 | H(31)-N(9)-C(10) | 116.2476 | H(24)-C(3)-H(23) | 108.8763 |
| H(36)-C(16)-C(15) | 120.0996 | H(31)-N(9)-N(8) | 110.8756 | H(24)-C(3)-H(22) | 108.2188 |
| C(17)-C(16)-C(15) | 120.1582 | C(10)-N(9)-N(8) | 118.6424 | H(24)-C(3)-N(2) | 111.6977 |
| H(35)-C(15)-C(16) | 119.7876 | H(30)-N(8)-N(9) | 114.2668 | H(23)-C(3)-H(22) | 109.0549 |
| H(35)-C(15)-C(14) | 120.4397 | H(30)-N(8)-C(6) | 114.6065 | H(23)-C(3)-N(2) | 109.5714 |
| C(16)-C(15)-C(14) | 119.7721 | N(9)-N(8)-C(6) | 118.8838 | H(22)-C(3)-N(2) | 109.3745 |
| H(34)-C(14)-C(15) | 119.9812 | N(8)-C(6)-O(7) | 114.6352 | C(5)-N(2)-C(4) | 99.9784 |
| H(34)-C(14)-C(13) | 119.1495 | N(8)-C(6)-C(5) | 120.7246 | C(5)-N(2)-C(3) | 100.2282 |
| C(15)-C(14)-C(13) | 120.8542 | O(7)-C(6)-C(5) | 124.6397 | C(5)-N(2)-C(1) | 100.2571 |
| H(33)-C(13)-C(14) | 120.9764 | H(29)-C(5)-H(28) | 119.4648 | C(4)-N(2)-C(3) | 117.0318 |
| H(33)-C(13)-C(12) | 119.7687 | H(29)-C(5)-C(6) | 117.6588 | C(4)-N(2)-C(1) | 116.8853 |
| C(14)-C(13)-C(12) | 119.2352 | H(29)-C(5)-N(2) | 74.9481 | C(3)-N(2)-C(1) | 116.9642 |
| C(17)-C(12)-C(13) | 120.0618 | H(28)-C(5)-C(6) | 121.4549 | H(21)-C(1)-H(20) | 108.5768 |
| C(17)-C(12)-N(11) | 118.7034 | H(28)-C(5)-N(2) | 80.0227 | H(21)-C(1)-H(19) | 108.8147 |
| C(13)-C(12)-N(11) | 121.1391 | C(6)-C(5)-N(2) | 103.492 | H(21)-C(1)-N(2) | 109.4205 |
| H(32)-N(11)-C(12) | 116.6736 | H(27)-C(4)-H(26) | 108.3651 | H(20)-C(1)-H(19) | 108.1179 |
| H(32)-N(11)-C(10) | 116.5169 | H(27)-C(4)-H(25) | 109.0283 | H(20)-C(1)-N(2) | 112.2797 |
| C(12)-N(11)-C(10) | 126.6408 | H(27)-C(4)-N(2) | 109.8632 | H(19)-C(1)-N(2) | 109.562 |

**Table (S4)**: Bonds lengths (Å) of Cu-L using DFT-method from DMOL^3^calculations

| **Bond** | **Length(Å)** | **Bond** | **Length(Å)** | **Bond** | **Length(Å)** | **Bond** | **Length(Å)** |
| --- | --- | --- | --- | --- | --- | --- | --- |
| S(16)-H(51) | 1.366 | C(19)-H(42) | 1.14 | C(11)-H(35) | 1.1362 | C(4)-O(5) | 1.521 |
| O(26)-H(27) | 1.1101 | C(19)-H(41) | 1.14 | C(11)-C(12) | 1.5401 | C(3)-H(32) | 1.1401 |
| C(25)-H(50) | 1.1396 | C(19)-H(40) | 1.1399 | C(15)-C(10) | 1.5415 | C(3)-H(31) | 1.14 |
| C(25)-H(49) | 1.1399 | O(26)-Cu(17) | 2.1155 | C(10)-C(11) | 1.5432 | C(3)-C(4) | 1.5401 |
| C(25)-O(26) | 1.5119 | O(21)-Cu(17) | 2.1149 | N(9)-H(34) | 1.11 | C(2)-H(30) | 1.14 |
| C(24)-H(48) | 1.1398 | Cl(18)-Cu(17) | 2.3625 | N(9)-C(10) | 1.5176 | C(2)-H(29) | 1.14 |
| C(24)-H(47) | 1.1394 | S(16)-Cu(17) | 2.3923 | C(8)-S(16) | 1.8754 | C(2)-H(28) | 1.1391 |
| C(24)-H(46) | 1.1399 | C(15)-H(39) | 1.1401 | C(8)-N(9) | 1.5168 | N(1)-C(20) | 1.5093 |
| C(24)-C(25) | 1.5411 | C(14)-H(38) | 1.14 | N(7)-Cu(17) | 2.0495 | N(1)-C(19) | 1.5096 |
| O(21)-H(23) | 1.1099 | C(14)-C(15) | 1.5398 | N(7)-C(8) | 1.5385 | N(1)-C(3) | 1.5108 |
| O(21)-H(22) | 1.11 | C(13)-H(37) | 1.14 | N(6)-H(33) | 1.11 | N(1)-C(2) | 1.5102 |
| C(20)-H(45) | 1.1401 | C(13)-C(14) | 1.5386 | N(6)-N(7) | 1.4846 |  |  |
| C(20)-H(44) | 1.14 | C(12)-H(36) | 1.14 | O(5)-Cu(17) | 2.1041 |  |  |
| C(20)-H(43) | 1.1402 | C(12)-C(13) | 1.5387 | C(4)-N(6) | 1.5296 |  |  |

**Table (S5): Bonds angles (°) of Cu-L1 using DFT-method from DMOL3 calculations**

| **Angle** | **Degree(°)** | **Angle** | **Degree(°)** | **Angle** | **Degree(°)** |
| --- | --- | --- | --- | --- | --- |
| H(27)-O(26)-C(25) | 110.0025 | O(26)-Cu(17)-O(5) | 91.8153 | C(10)-N(9)-C(8) | 122.8051 |
| H(27)-O(26)-Cu(17) | 108.9873 | O(21)-Cu(17)-Cl(18) | 89.2636 | S(16)-C(8)-N(9) | 124.7275 |
| C(25)-O(26)-Cu(17) | 113.7996 | O(21)-Cu(17)-S(16) | 88.6092 | S(16)-C(8)-N(7) | 112.394 |
| H(50)-C(25)-H(49) | 109.0054 | O(21)-Cu(17)-N(7) | 92.8533 | N(9)-C(8)-N(7) | 122.0692 |
| H(50)-C(25)-O(26) | 110.0836 | O(21)-Cu(17)-O(5) | 177.638 | Cu(17)-N(7)-C(8) | 92.7854 |
| H(50)-C(25)-C(24) | 110.2199 | Cl(18)-Cu(17)-S(16) | 173.9936 | Cu(17)-N(7)-N(6) | 111.0622 |
| H(49)-C(25)-O(26) | 108.5635 | Cl(18)-Cu(17)-N(7) | 95.3198 | C(8)-N(7)-N(6) | 117.2143 |
| H(49)-C(25)-C(24) | 108.3149 | Cl(18)-Cu(17)-O(5) | 89.6402 | H(33)-N(6)-N(7) | 122.5694 |
| O(26)-C(25)-C(24) | 110.601 | S(16)-Cu(17)-N(7) | 79.1799 | H(33)-N(6)-C(4) | 122.5088 |
| H(48)-C(24)-H(47) | 109.2584 | S(16)-Cu(17)-O(5) | 92.2755 | N(7)-N(6)-C(4) | 114.6337 |
| H(48)-C(24)-H(46) | 109.4572 | N(7)-Cu(17)-O(5) | 85.1652 | Cu(17)-O(5)-C(4) | 108.5454 |
| H(48)-C(24)-C(25) | 109.3128 | H(51)-S(16)-Cu(17) | 39.3615 | N(6)-C(4)-O(5) | 115.6276 |
| H(47)-C(24)-H(46) | 109.186 | H(51)-S(16)-C(8) | 57.7732 | N(6)-C(4)-C(3) | 122.1632 |
| H(47)-C(24)-C(25) | 110.1353 | Cu(17)-S(16)-C(8) | 74.7092 | O(5)-C(4)-C(3) | 122.2084 |
| H(46)-C(24)-C(25) | 109.4763 | H(39)-C(15)-C(14) | 119.8301 | H(32)-C(3)-H(31) | 109.3922 |
| H(23)-O(21)-H(22) | 109.4921 | H(39)-C(15)-C(10) | 119.8458 | H(32)-C(3)-C(4) | 109.0053 |
| H(23)-O(21)-Cu(17) | 109.5111 | C(14)-C(15)-C(10) | 120.3208 | H(32)-C(3)-N(1) | 109.1699 |
| H(22)-O(21)-Cu(17) | 109.3389 | H(38)-C(14)-C(15) | 119.9992 | H(31)-C(3)-C(4) | 109.5361 |
| H(45)-C(20)-H(44) | 109.5608 | H(38)-C(14)-C(13) | 120.0116 | H(31)-C(3)-N(1) | 109.4162 |
| H(45)-C(20)-H(43) | 109.5624 | C(15)-C(14)-C(13) | 119.9888 | C(4)-C(3)-N(1) | 110.3038 |
| H(45)-C(20)-N(1) | 109.4988 | H(37)-C(13)-C(14) | 120.0532 | H(30)-C(2)-H(29) | 109.4709 |
| H(44)-C(20)-H(43) | 109.527 | H(37)-C(13)-C(12) | 120.0408 | H(30)-C(2)-H(28) | 109.206 |
| H(44)-C(20)-N(1) | 109.4492 | C(14)-C(13)-C(12) | 119.9031 | H(30)-C(2)-N(1) | 109.3036 |
| H(43)-C(20)-N(1) | 109.2287 | H(36)-C(12)-C(13) | 119.9515 | H(29)-C(2)-H(28) | 109.605 |
| H(42)-C(19)-H(41) | 109.4955 | H(36)-C(12)-C(11) | 119.946 | H(29)-C(2)-N(1) | 109.4584 |
| H(42)-C(19)-H(40) | 109.5007 | C(13)-C(12)-C(11) | 120.0996 | H(28)-C(2)-N(1) | 109.7826 |
| H(42)-C(19)-N(1) | 109.4671 | H(35)-C(11)-C(12) | 119.461 | C(20)-N(1)-C(19) | 109.7565 |
| H(41)-C(19)-H(40) | 109.491 | H(35)-C(11)-C(10) | 120.3801 | C(20)-N(1)-C(3) | 109.2004 |
| H(41)-C(19)-N(1) | 109.4639 | C(12)-C(11)-C(10) | 120.1401 | C(20)-N(1)-C(2) | 109.1954 |
| H(40)-C(19)-N(1) | 109.4091 | C(15)-C(10)-C(11) | 119.4375 | C(19)-N(1)-C(3) | 109.0773 |
| O(26)-Cu(17)-O(21) | 90.2892 | C(15)-C(10)-N(9) | 118.6452 | C(19)-N(1)-C(2) | 109.0399 |
| O(26)-Cu(17)-Cl(18) | 90.6511 | C(11)-C(10)-N(9) | 121.901 | C(3)-N(1)-C(2) | 110.559 |
| O(26)-Cu(17)-S(16) | 94.9756 | H(34)-N(9)-C(10) | 118.5867 |  |  |
| O(26)-Cu(17)-N(7) | 173.2842 | H(34)-N(9)-C(8) | 118.5981 |  |  |

**Table (S 6)**: Bonds lengths (Å) of Co-L using DFT-method from DMOL^3^ calculations

| **Bond** | **Length(Å)** | **Bond** | **Length(Å)** | **Bond** | **Length(Å)** | **Bond** | **Length(Å)** |
| --- | --- | --- | --- | --- | --- | --- | --- |
| S(16)-H(58) | 1.366 | O(25)-C(26) | 1.5118 | C(15)-H(40) | 1.14 | N(7)-Co(17) | 2.0535 |
| C(31)-H(57) | 1.1405 | O(23)-H(24) | 1.1101 | C(14)-H(39) | 1.1401 | N(7)-C(8) | 1.5384 |
| C(31)-H(56) | 1.1398 | C(22)-H(45) | 1.14 | C(14)-C(15) | 1.539 | N(6)-H(34) | 1.1098 |
| C(31)-H(55) | 1.14 | C(22)-H(44) | 1.1401 | C(13)-H(38) | 1.14 | N(6)-N(7) | 1.482 |
| C(30)-H(54) | 1.1399 | C(22)-O(23) | 1.5098 | C(13)-C(14) | 1.5378 | O(5)-Co(17) | 2.1088 |
| C(30)-H(53) | 1.14 | C(21)-H(43) | 1.1401 | C(12)-H(37) | 1.1399 | C(4)-N(6) | 1.5296 |
| C(30)-H(52) | 1.1401 | C(21)-H(42) | 1.1401 | C(12)-C(13) | 1.5383 | C(4)-O(5) | 1.5233 |
| C(29)-H(51) | 1.1398 | C(21)-H(41) | 1.14 | C(11)-H(36) | 1.1337 | C(3)-H(33) | 1.14 |
| C(29)-H(50) | 1.1399 | C(21)-C(22) | 1.5402 | C(11)-C(12) | 1.5408 | C(3)-H(32) | 1.14 |
| C(29)-H(49) | 1.1402 | O(18)-H(20) | 1.1104 | C(15)-C(10) | 1.5417 | C(3)-C(4) | 1.5415 |
| C(28)-H(48) | 1.14 | O(18)-H(19) | 1.1096 | C(10)-C(11) | 1.5452 | N(1)-C(31) | 1.7103 |
| C(28)-H(47) | 1.1401 | O(25)-Co(17) | 2.1204 | N(9)-H(35) | 1.1101 | N(1)-C(30) | 1.7106 |
| C(28)-H(46) | 1.1399 | O(23)-Co(17) | 2.1192 | N(9)-C(10) | 1.5205 | C(29)-N(1) | 1.7141 |
| C(26)-C(28) | 1.5404 | O(18)-Co(17) | 2.1221 | C(8)-S(16) | 1.8765 | N(1)-C(3) | 1.7129 |
| C(26)-O(27) | 1.5104 | S(16)-Co(17) | 2.396 | C(8)-N(9) | 1.52 | N(1)-Cl(2) | 1.9236 |

**Table (7S)**: Bonds angles (°) of Co-L using DFT-method from DMOL^3^ calculations

| **Angle** | **Degree(°)** | **Angle** | **Degree(°)** | **Angle** | **Degree(°)** |
| --- | --- | --- | --- | --- | --- |
| H(57)-C(31)-H(56) | 109.4184 | H(43)-C(21)-H(41) | 109.4924 | H(36)-C(11)-C(12) | 119.1574 |
| H(57)-C(31)-H(55) | 109.6042 | H(43)-C(21)-C(22) | 109.4207 | H(36)-C(11)-C(10) | 120.6581 |
| H(57)-C(31)-N(1) | 109.3543 | H(42)-C(21)-H(41) | 109.5189 | C(12)-C(11)-C(10) | 120.1744 |
| H(56)-C(31)-H(55) | 109.5614 | H(42)-C(21)-C(22) | 109.3938 | C(15)-C(10)-C(11) | 119.2537 |
| H(56)-C(31)-N(1) | 109.4305 | H(41)-C(21)-C(22) | 109.6098 | C(15)-C(10)-N(9) | 117.7953 |
| H(55)-C(31)-N(1) | 109.4582 | H(20)-O(18)-H(19) | 109.765 | C(11)-C(10)-N(9) | 122.9471 |
| H(54)-C(30)-H(53) | 109.5135 | H(20)-O(18)-Co(17) | 109.118 | H(35)-N(9)-C(10) | 117.8789 |
| H(54)-C(30)-H(52) | 109.4362 | H(19)-O(18)-Co(17) | 109.5895 | H(35)-N(9)-C(8) | 117.8815 |
| H(54)-C(30)-N(1) | 109.4896 | O(25)-Co(17)-O(23) | 90.4386 | C(10)-N(9)-C(8) | 124.2308 |
| H(53)-C(30)-H(52) | 109.5014 | O(25)-Co(17)-O(18) | 89.7276 | S(16)-C(8)-N(9) | 126.0344 |
| H(53)-C(30)-N(1) | 109.5328 | O(25)-Co(17)-S(16) | 88.7707 | S(16)-C(8)-N(7) | 112.3349 |
| H(52)-C(30)-N(1) | 109.3536 | O(25)-Co(17)-N(7) | 93.2037 | N(9)-C(8)-N(7) | 121.5658 |
| H(51)-C(29)-H(50) | 109.512 | O(25)-Co(17)-O(5) | 177.6645 | Co(17)-N(7)-C(8) | 92.9844 |
| H(51)-C(29)-H(49) | 109.4716 | O(23)-Co(17)-O(18) | 90.1223 | Co(17)-N(7)-N(6) | 111.2379 |
| H(51)-C(29)-N(1) | 109.6018 | O(23)-Co(17)-S(16) | 95.4076 | C(8)-N(7)-N(6) | 117.0204 |
| H(50)-C(29)-H(49) | 109.4311 | O(23)-Co(17)-N(7) | 173.2569 | H(34)-N(6)-N(7) | 122.4606 |
| H(50)-C(29)-N(1) | 109.4464 | O(23)-Co(17)-O(5) | 91.6302 | H(34)-N(6)-C(4) | 122.5529 |
| H(49)-C(29)-N(1) | 109.3642 | O(18)-Co(17)-S(16) | 174.28 | N(7)-N(6)-C(4) | 114.7748 |
| H(48)-C(28)-H(47) | 109.4625 | O(18)-Co(17)-N(7) | 95.5637 | Co(17)-O(5)-C(4) | 108.1224 |
| H(48)-C(28)-H(46) | 109.4434 | O(18)-Co(17)-O(5) | 89.1844 | N(6)-C(4)-O(5) | 115.5455 |
| H(48)-C(28)-C(26) | 109.6826 | S(16)-Co(17)-N(7) | 79.0132 | N(6)-C(4)-C(3) | 122.5003 |
| H(47)-C(28)-H(46) | 109.3705 | S(16)-Co(17)-O(5) | 92.1124 | O(5)-C(4)-C(3) | 121.9532 |
| H(47)-C(28)-C(26) | 109.5617 | N(7)-Co(17)-O(5) | 84.8458 | H(33)-C(3)-H(32) | 109.4779 |
| H(46)-C(28)-C(26) | 109.3061 | H(58)-S(16)-Co(17) | 34.3952 | H(33)-C(3)-C(4) | 108.2699 |
| C(28)-C(26)-O(27) | 119.7989 | H(58)-S(16)-C(8) | 55.8509 | H(33)-C(3)-N(1) | 108.7347 |
| C(28)-C(26)-O(25) | 119.9158 | Co(17)-S(16)-C(8) | 74.8543 | H(32)-C(3)-C(4) | 111.0717 |
| O(27)-C(26)-O(25) | 120.1271 | H(40)-C(15)-C(14) | 119.7375 | H(32)-C(3)-N(1) | 110.215 |
| C(26)-O(25)-Co(17) | 110.3204 | H(40)-C(15)-C(10) | 119.7461 | C(4)-C(3)-N(1) | 109.0165 |
| H(24)-O(23)-C(22) | 109.4402 | C(14)-C(15)-C(10) | 120.516 | C(31)-N(1)-C(30) | 118.5587 |
| H(24)-O(23)-Co(17) | 109.6073 | H(39)-C(14)-C(15) | 119.9964 | C(31)-N(1)-C(29) | 90.0688 |
| C(22)-O(23)-Co(17) | 109.9797 | H(39)-C(14)-C(13) | 120.0059 | C(31)-N(1)-C(3) | 92.451 |
| H(45)-C(22)-H(44) | 109.4492 | C(15)-C(14)-C(13) | 119.9976 | C(31)-N(1)-Cl(2) | 121.3314 |
| H(45)-C(22)-O(23) | 109.5682 | H(38)-C(13)-C(14) | 120.0604 | C(30)-N(1)-C(29) | 89.8175 |
| H(45)-C(22)-C(21) | 109.6999 | H(38)-C(13)-C(12) | 120.074 | C(30)-N(1)-C(3) | 92.8255 |
| H(44)-C(22)-O(23) | 109.501 | C(14)-C(13)-C(12) | 119.8653 | C(30)-N(1)-Cl(2) | 119.7964 |
| H(44)-C(22)-C(21) | 109.6635 | H(37)-C(12)-C(13) | 119.9138 | C(29)-N(1)-C(3) | 174.9412 |
| O(23)-C(22)-C(21) | 108.9448 | H(37)-C(12)-C(11) | 119.9139 | C(29)-N(1)-Cl(2) | 84.6082 |
| H(43)-C(21)-H(42) | 109.3914 | C(13)-C(12)-C(11) | 120.1712 | C(3)-N(1)-Cl(2) | 90.3349 |

**Table (S 8)**: Bonds lengths (Å) of Ni-L using DFT-method from DMOL^3^ calculations.

| **Bond** | **Length (Å)** | **Bond** | **Length (Å)** | **Bond** | **Length (Å)** | **Bond** | **Length (Å)** |
| --- | --- | --- | --- | --- | --- | --- | --- |
| S(16)-H(47) | 1.366 | C(18)-H(37) | 1.1398 | C(15)-C(10) | 1.5397 | C(3)-H(28) | 1.1396 |
| O(22)-H(23) | 1.1102 | C(18)-H(36) | 1.1399 | C(10)-C(11) | 1.5405 | C(3)-H(27) | 1.1402 |
| C(21)-H(46) | 1.1399 | O(22)-Ni(17) | 1.9491 | N(9)-H(30) | 1.1098 | C(3)-C(4) | 1.5369 |
| C(21)-H(45) | 1.1396 | S(16)-Ni(17) | 2.2912 | N(9)-C(10) | 1.5124 | C(2)-H(26) | 1.1402 |
| C(21)-H(44) | 1.1398 | C(15)-H(35) | 1.1387 | C(8)-S(16) | 1.804 | C(2)-H(25) | 1.1398 |
| C(20)-H(43) | 1.1398 | C(14)-H(34) | 1.14 | C(8)-N(9) | 1.5144 | C(2)-H(24) | 1.1402 |
| C(20)-H(42) | 1.1401 | C(14)-C(15) | 1.5401 | N(7)-Ni(17) | 1.9381 | N(1)-C(19) | 1.5102 |
| C(20)-O(22) | 1.5119 | C(13)-H(33) | 1.1398 | N(7)-C(8) | 1.4712 | N(1)-C(18) | 1.5097 |
| C(20)-C(21) | 1.5418 | C(13)-C(14) | 1.5392 | N(6)-H(29) | 1.1095 | N(1)-C(3) | 1.5099 |
| C(19)-H(41) | 1.1399 | C(12)-H(32) | 1.1401 | N(6)-N(7) | 1.4663 | N(1)-C(2) | 1.5107 |
| C(19)-H(40) | 1.1397 | C(12)-C(13) | 1.5395 | O(5)-Ni(17) | 1.9604 |  |  |
| C(19)-H(39) | 1.1407 | C(11)-H(31) | 1.1401 | C(4)-N(6) | 1.4996 |  |  |
| C(18)-H(38) | 1.1401 | C(11)-C(12) | 1.5407 | C(4)-O(5) | 1.5141 |  |  |

**Table (S9):**  Bonds angles (°) of Ni-L using DFT-method from DMOL^3^ calculations.

| **Angle** | **Degree (°)** | **Angle** | **Degree (°)** | **Angle** | **Degree (°)** |
| --- | --- | --- | --- | --- | --- |
| H(23)-O(22)-C(20) | 110.0248 | S(16)-Ni(17)-N(7) | 74.0876 | Ni(17)-N(7)-C(8) | 102.6599 |
| H(23)-O(22)-Ni(17) | 109.3526 | S(16)-Ni(17)-O(5) | 134.208 | Ni(17)-N(7)-N(6) | 117.2693 |
| C(20)-O(22)-Ni(17) | 110.1962 | N(7)-Ni(17)-O(5) | 84.4827 | C(8)-N(7)-N(6) | 128.8662 |
| H(46)-C(21)-H(45) | 108.9368 | H(47)-S(16)-Ni(17) | 52.3933 | H(29)-N(6)-N(7) | 124.5913 |
| H(46)-C(21)-H(44) | 109.3345 | H(47)-S(16)-C(8) | 29.5431 | H(29)-N(6)-C(4) | 124.3553 |
| H(46)-C(21)-C(20) | 109.4084 | Ni(17)-S(16)-C(8) | 80.7166 | N(7)-N(6)-C(4) | 110.9071 |
| H(45)-C(21)-H(44) | 109.3085 | H(35)-C(15)-C(14) | 119.7654 | Ni(17)-O(5)-C(4) | 113.1169 |
| H(45)-C(21)-C(20) | 110.47 | H(35)-C(15)-C(10) | 119.9904 | N(6)-C(4)-O(5) | 113.21 |
| H(44)-C(21)-C(20) | 109.364 | C(14)-C(15)-C(10) | 120.2056 | N(6)-C(4)-C(3) | 123.4583 |
| H(43)-C(20)-H(42) | 109.3325 | H(34)-C(14)-C(15) | 119.9824 | O(5)-C(4)-C(3) | 123.3255 |
| H(43)-C(20)-O(22) | 108.7979 | H(34)-C(14)-C(13) | 120.0477 | H(28)-C(3)-H(27) | 110.147 |
| H(43)-C(20)-C(21) | 108.9084 | C(15)-C(14)-C(13) | 119.9665 | H(28)-C(3)-C(4) | 110.3124 |
| H(42)-C(20)-O(22) | 109.5332 | H(33)-C(13)-C(14) | 119.9966 | H(28)-C(3)-N(1) | 110.2787 |
| H(42)-C(20)-C(21) | 109.6715 | H(33)-C(13)-C(12) | 120.0373 | H(27)-C(3)-C(4) | 109.8571 |
| O(22)-C(20)-C(21) | 110.5734 | C(14)-C(13)-C(12) | 119.9653 | H(27)-C(3)-N(1) | 110.1895 |
| H(41)-C(19)-H(40) | 109.5559 | H(32)-C(12)-C(13) | 120.0048 | C(4)-C(3)-N(1) | 105.9794 |
| H(41)-C(19)-H(39) | 109.5353 | H(32)-C(12)-C(11) | 120.0101 | H(26)-C(2)-H(25) | 109.5229 |
| H(41)-C(19)-N(1) | 109.4426 | C(13)-C(12)-C(11) | 119.9851 | H(26)-C(2)-H(24) | 109.4189 |
| H(40)-C(19)-H(39) | 109.3998 | H(31)-C(11)-C(12) | 119.8921 | H(26)-C(2)-N(1) | 109.4605 |
| H(40)-C(19)-N(1) | 109.4829 | H(31)-C(11)-C(10) | 119.9641 | H(25)-C(2)-H(24) | 109.5041 |
| H(39)-C(19)-N(1) | 109.4106 | C(12)-C(11)-C(10) | 120.1435 | H(25)-C(2)-N(1) | 109.4492 |
| H(38)-C(18)-H(37) | 109.4244 | C(15)-C(10)-C(11) | 119.7022 | H(24)-C(2)-N(1) | 109.4716 |
| H(38)-C(18)-H(36) | 109.402 | C(15)-C(10)-N(9) | 120.5141 | C(19)-N(1)-C(18) | 109.8403 |
| H(38)-C(18)-N(1) | 109.6783 | C(11)-C(10)-N(9) | 119.6375 | C(19)-N(1)-C(3) | 108.6804 |
| H(37)-C(18)-H(36) | 109.468 | H(30)-N(9)-C(10) | 119.0867 | C(19)-N(1)-C(2) | 109.1716 |
| H(37)-C(18)-N(1) | 109.3888 | H(30)-N(9)-C(8) | 119.225 | C(18)-N(1)-C(3) | 110.5071 |
| H(36)-C(18)-N(1) | 109.4657 | C(10)-N(9)-C(8) | 121.6486 | C(18)-N(1)-C(2) | 109.7776 |
| O(22)-Ni(17)-S(16) | 113.6844 | S(16)-C(8)-N(9) | 126.8438 | C(3)-N(1)-C(2) | 108.8334 |
| O(22)-Ni(17)-N(7) | 109.9047 | S(16)-C(8)-N(7) | 102.5356 |  |  |
| O(22)-Ni(17)-O(5) | 111.5552 | N(9)-C(8)-N(7) | 129.7134 |  |  |
